# The New Paradigm of Ligand Substitution-Driven Enhancement of Anisotropy from SO_4_ Units in Short-Wavelength Region

**DOI:** 10.1021/acscentsci.4c01401

**Published:** 2024-11-27

**Authors:** Chenhui Hu, Huimin Li, Guangsheng Xu, Zhihua Yang, Jian Han, Shilie Pan

**Affiliations:** † Research Center for Crystal Materials, State Key Laboratory of Functional Materials and Devices for Special Environmental Conditions, Xinjiang Key Laboratory of Functional Crystal Materials, Xinjiang Technical Institute of Physics and Chemistry, CAS, 40-1 South Beijing Road, Urumqi 830011, China; ‡ Center of Materials Science and Optoelectronics Engineering, University of Chinese Academy of Sciences, Beijing 100049, China

## Abstract

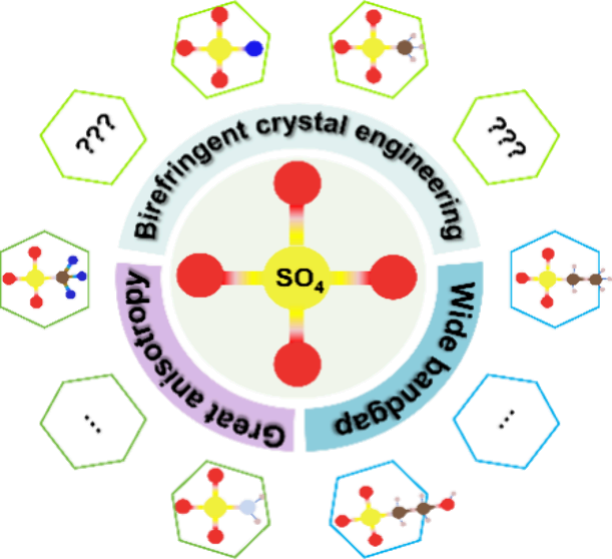

For non-π-conjugated [SO_4_] units, it is challenging to generate sufficient birefringence, owing to the high symmetry of the regular tetrahedron. Unlike the traditional trial-and-error approach, we propose a new paradigm for birefringence engineering to tune the optical properties based on [SO_4_] units. Through the strategy of ligand substitution, we can predict its effect on the band gap and anisotropy. Theoretical evaluations reveal generalized results that the anisotropic electron distribution of new functional groups induced by the suitable ligand substitution contributes to the band gap and birefringence. To further validate the correctness of the paradigm, we experimentally synthesized and characterized nine novel compounds with selected functional modules. By the new paradigm of ligand substitution, they can reach up to 4–6 times the birefringence of the corresponding sulfate and maintain the wide bandgap. Through rational design, (CN_4_H_7_)SO_3_NH_2_ exhibits about 35 times the birefringence of Li_2_SO_4_, which is a significant order of magnitude improvement and verifies the superiority of our proposed paradigm. This work provides a new paradigm for the modification to the non-π-conjugated group and will guide and accelerate the exploration of novel birefringent crystals in the short-wavelength region.

## Abstract 2

The new paradigm of ligand substitution focusing on [SO_4_] units is proposed and utilized to fine-tune the optical anisotropy, which guides the discovery of novel materials with great birefringence.

## Introduction

Birefringent materials can split incident light into two refracted rays, manipulating the polarization of the light. In recent years, ultraviolet (UV) materials with large birefringence have been developed due to the rapid progress of UV light sources in various fields.^1−8^ Many strategies and efforts have been employed in the design of materials with great birefringence in short-wavelength regions (especially at around and below 200 nm). Research on novel birefringent materials has focused on the field of material systems with π-conjugated groups, such as [BO_3_], [CO_3_], and [NO_3_] units. However, this has not led to a wider transparency window due to π-conjugation effects.^9−22^ Non-π-conjugated [SO_4_] tetrahedra are not of interest as birefringent groups due to weak optical anisotropy. Synthesized crystals containing non-π-conjugated [SO_4_] groups typically exhibit large bandgaps but small birefringence, making them difficult to satisfy practical needs.^23−26^ Therefore, it is essential to significantly improve the birefringence of the compounds containing [SO_4_] tetrahedra. Birefringence enhancement induced by new functional modules has been a hot topic in materials science for a long time.^27−29^

To enhance the birefringence of crystals with non-π-conjugated groups, many methods have been tried. Introducing d^0^ transition metals with the distortions of second-order Jahn–Teller (SOJT), or metal cations with lone pairs, such as Sb^3+^, Bi^3+^, Pb^2+^, and Sn^2+^, can also produce a large birefringence.^30−40^ Although these are high-quality strategies, it is crucial to acknowledge that crystals with stereochemical activity or transition metals may result in a narrow band gap, making them unsuitable for the short-wavelength region application.^41−45^ Recently, a design strategy about birefringent materials was proposed, which utilizes the functions of cation groups to enhance birefringence, breaking the dominance of anions in the design of UV optical materials with large birefringence.^46^ Additionally, combining non-π-conjugated groups and π-conjugated [CO_3_] and [NO_3_] groups is expected to enhance the birefringence.^47^

Nonetheless, it does not fundamentally alter the [SO_4_] tetrahedra, and the issue of their weak anisotropy remains unaddressed. A more direct approach is proposed to solve the small anisotropy of non-π-conjugated groups. In our group’s previous study, the fluorination idea was proposed as a powerful strategy for the chemical and functional modification of materials.^48−58^ Numerous exceptional and remarkable treasure crystals have been discovered, capturing the interest of scientists worldwide.

The integration of new chemical modules into these tetrahedra has been effective, particularly through the partial decoration of the oxygen with other modules, which is called ligand substitution. However, targeted substitution remains challenging due to the complexities arising from the differences in atomic properties, such as radius, charge, and electronegativity, which can disrupt the symmetry of the original tetrahedron and alter the electron cloud distribution. Recently, our group designed the [B(OH)_3_CH_3_] unit, which showcases enhanced polarizability anisotropy and can serve as active fundamental building blocks (FBBs) in birefringence.^59^ Furthermore, there is a need for more and better birefringent active modules with a wide bandgap based on tetrahedra.^60−65^

In addition, irrational ligands may negatively impact the optical properties, making it crucial to select ligands carefully. π-conjugated ligands with significant anisotropy are excellent candidates for designing crystals with a high degree of birefringence, but they can cause irreversible effects on the band gap. To significantly improve the anisotropy of [SO_4_] units, we introduced heteroatoms or non-π-conjugated long chains in highly symmetric tetrahedra. Drawing upon this insight, some derived systems were obtained, including [SO_3_F], [SO_4_F], [SO_3_NH_2_], [SO_4_NH_3_], [SO_2_(NH_2_)_2_], [SOClCH_3_], [SO_3_S], [SO_3_CH_3_], [SO_3_C_2_H_5_], [SO_3_C_2_H_4_OH], [SO(CH_3_)_2_NH], and [SO_2_CH_3_NH_2_] groups, which can be considered as the novel groups in the design of birefringent materials.

In this work, we obtained the crystal structures of 15 compounds to verify the correctness of our birefringent engineering strategy by ligand substitution, of which the structures of nine new crystals were reported by us for the first time, including LiSO_3_C_2_H_4_OH, LiSO_3_C_2_H_5_·H_2_O, NaSO_3_C_2_H_4_OH, KSO_3_C_2_H_4_OH, CsH(SO_3_CH_3_)_2_, (CN_4_H_7_)SO_3_NH_2_, (NH_3_OH)SO_3_CH_3_, Zn(SO_3_C_2_H_5_)_2_·6H_2_O, and (CN_4_H_7_)S_2_O_3_. Both theoretical simulations and experimental results demonstrate that the birefringence of these crystals with well-designed birefringent-active modular structures is significantly greater than that of the corresponding sulfate, which confirms the effectiveness of the proposed strategy. We aimed to enhance the birefringence of sulfate-derived materials significantly by strategically tailoring the valuable [SO_4_] groups and implementing ligand substitution.

## Results and Discussion

### Modification of [SO_4_] Group

**Figure 1:**
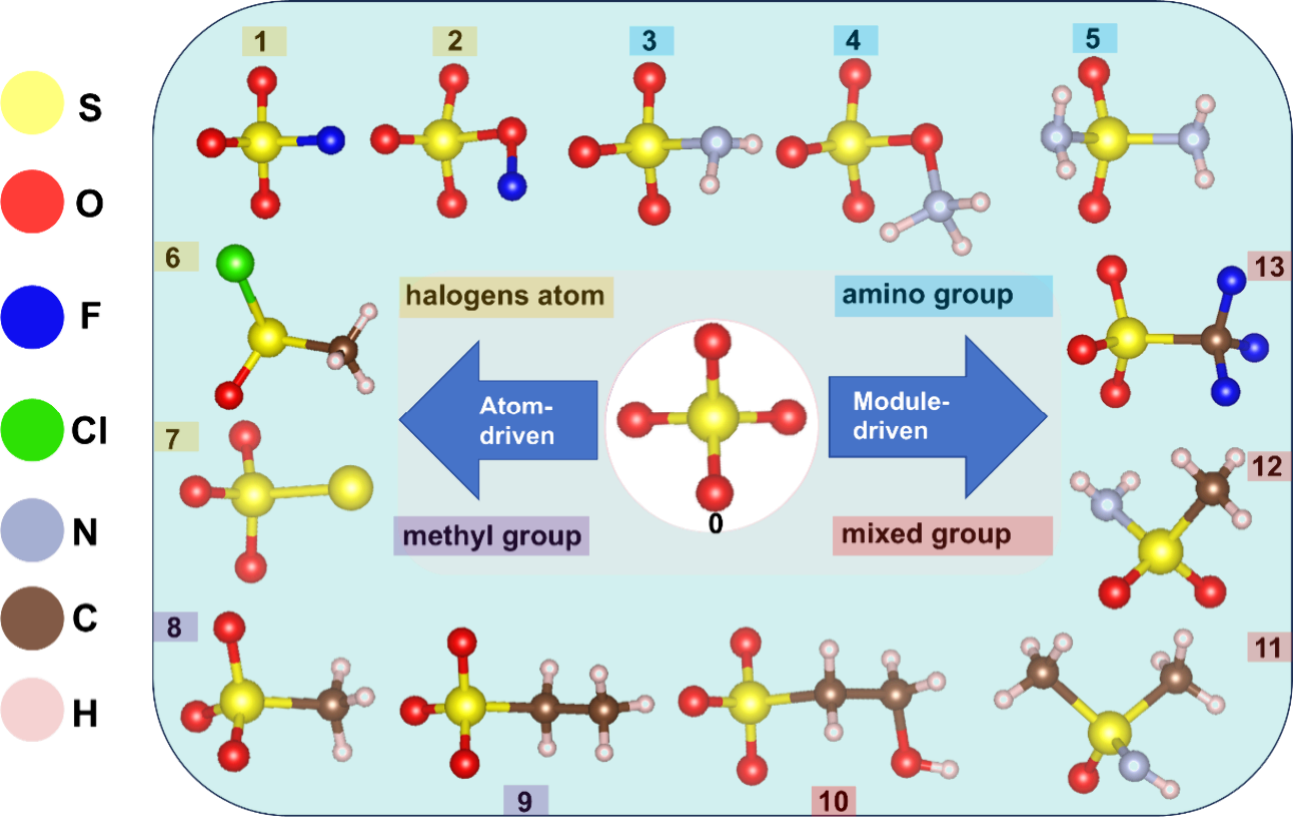
Exploration of crystal engineering modifications based on [SO_4_](0) units, including [SO_3_F](1), [SO_4_F](2), [SO_3_NH_2_](3), [SO_4_NH_3_](4), [SO_2_(NH_2_)_2_](5), [SOCl(CH_3_)](6), [SO_3_S](7), [SO_3_CH_3_](8), [SO_3_C_2_H_5_](9), [SO_3_C_2_H_4_OH](10), [SO(CH_3_)_2_NH](11), [SO_2_CH_3_NH_2_](12), and [SO_3_CF_3_](13) units.

To increase the anisotropy of the [SO_4_] group, various groups were attempted to be introduced to implement ligand substitution. The ligand substitution of [SO_4_] units through heteroatoms, e.g., halogen atoms, results in the [SO_3_F], [SO_4_F], [SOCl(CH_3_)], and [SO_3_S] groups. By introduction of amino groups, [SO_3_NH_2_], [SO_4_NH_3_], and [SO_2_(NH_2_)_2_] groups are tracked and selected. The alkyl group is introduced with a substituted oxygen atom, e.g., methyl, and [SO_3_CH_3_] and [SO_3_C_2_H_5_] groups are obtained. Through the synergistic action of mixed motifs, [SO_3_C_2_H_4_OH], [SO(CH_3_)_2_NH], [SO_2_CH_3_NH_2_], and [SO_3_CF_3_] groups are selected. Based on [SO_4_](0) units, we obtain and mark the [SO_3_F](1), [SO_4_F](2), [SO_3_NH_2_](3), [SO_4_NH_3_](4), [SO_2_(NH_2_)_2_](5), [SOCl(CH_3_)](6), [SO_3_S](7), [SO_3_CH_3_](8), [SO_3_C_2_H_5_](9), [SO_3_C_2_H_4_OH](10), [SO(CH_3_)_2_NH](11), [SO_2_CH_3_NH_2_](12) and [SO_3_CF_3_](13) groups from known structure by ligand substitution (Figure 1). When these groups were modified at the microscopic level, their impacts on the optical properties were preliminarily evaluated, which mainly focused on the anisotropy and energy gap.

**Figure 2:**
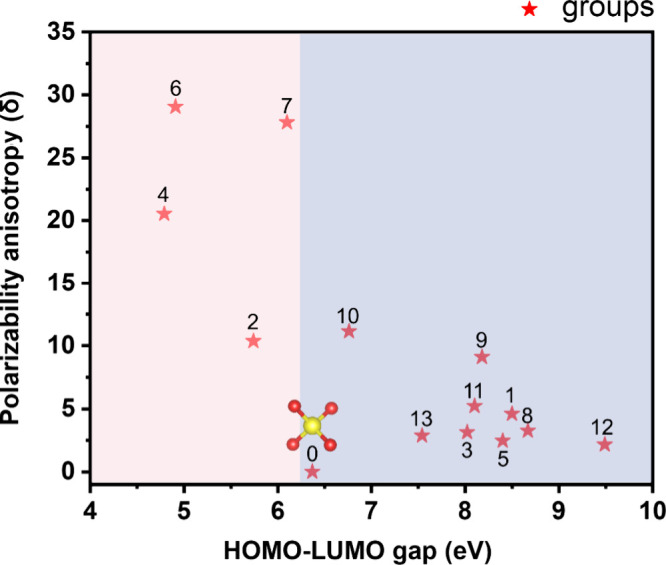
Comparison of the HOMO–LUMO gap and polarizability anisotropy for the groups by ligand substitution, where the groups have been numbered 0–13.

The orbital spatial distributions of the highest occupied molecular orbital (HOMO) and the lowest unoccupied molecular orbital (LUMO) gaps and the polarizability anisotropy of 14 groups were calculated by using DFT implemented by the Gaussian09 package. In comparison to our crystal structure template [SO_4_] units, which require modification, the anisotropy of the novel modules is improved to varying degrees, aligning with our goal in crystal engineering design (Figure 2). Developing excellent birefringent crystals with a wide bandgap is usually challenging due to the trade-off between bandgap and birefringence.

To explore birefringent crystals with wide bandgaps, the relevant optical properties of the groups theoretically are compared for selecting the superior groups. By excluding the groups with microscopic energy gaps below 6.2 eV, the designed materials can keep a wide transmission band window. The synthesized primitives have demonstrated excellent performance, such as [SO_3_F], [SO_3_NH_2_], [SO_3_CH_3_], and [SO_3_CF_3_] units.^44,49,65,66^ Compared with the [SO_4_] groups, the anisotropy of all the novel modules is enhanced (Figure 2). Introducing other elements or groups can break and optimize the configuration of the tetrahedra, making their symmetry decrease to *C*_3*v*_ or *C*_2*v*_, which further increases the polarizability anisotropy. Simultaneously, the newly proposed [SO_3_C_2_H_5_] and [SO_3_C_2_H_4_OH] groups exhibit significant anisotropy, which is greater than that of the [SO_3_F], [SO_3_NH_2_], [SO_3_CH_3_], and [SO_3_CF_3_] groups derived from [SO_4_] groups. In theory, materials constructed using these FBBs will exhibit substantial birefringence. To better validate the superiority of designed groups based on crystal birefringent engineering, we assembled them to obtain actual compounds for evaluating their optical properties. The crystal structures of 15 compounds were obtained, including LiSO_3_C_2_H_4_OH, LiSO_3_C_2_H_5_·H_2_O, NaSO_3_C_2_H_4_OH, KSO_3_C_2_H_4_OH, CsH(SO_3_CH_3_)_2_, (CN_4_H_7_)SO_3_NH_2_, Mg(SO_3_CH_3_)_2_·2H_2_O, Mn(SO_3_CH_3_)_2_·2H_2_O, NH_4_SO_3_CH_3_, Sr(SO_3_CH_3_)_2_·H_2_O, Zn(SO_3_CH_3_)_2_·4H_2_O, Zn(SO_3_C_2_H_4_OH)_2_·2H_2_O, (NH_3_OH)SO_3_CH_3_, Zn(SO_3_C_2_H_5_)_2_·6H_2_O, and (CN_4_H_7_)S_2_O_3_.

### Crystal Structure Description

Compounds Zn(SO_3_CH_3_)_2_·4H_2_O, Zn(SO_3_C_2_H_4_OH)_2_·2H_2_O, (NH_3_OH)SO_3_CH_3_, Zn(SO_3_C_2_H_5_)_2_·6H_2_O, and LiSO_3_C_2_H_5_·H_2_O crystallize in the same space group *P*2_1_*/c* (No. 14). Compounds KSO_3_C_2_H_4_OH, Mg(SO_3_CH_3_)_2_·2H_2_O, and Mn(SO_3_CH_3_)_2_·2H_2_O crystallize in space group *P*1̅. NH_4_SO_3_CH_3_ and (CN_4_H_7_)S_2_O_3_ crystallize in the same space group *C*2/*m*_._ Both NaSO_3_C_2_H_4_OH and CsH(SO_3_CH_3_)_2_ crystallize in space group *C*2*/c*. Sr(SO_3_CH_3_)_2_·H_2_O crystallizes in the monoclinic space group *P*2_1_/*m*, and LiSO_3_C_2_H_4_OH crystallizes in the orthogonal space group *Pbcn*. (CN_4_H_7_)SO_3_NH_2_ crystallizes in noncentrosymmetric space group *Pca*2_1_. More detailed structural information can be viewed in Tables S1–S4 in the Supporting Information.

**Figure 3:**
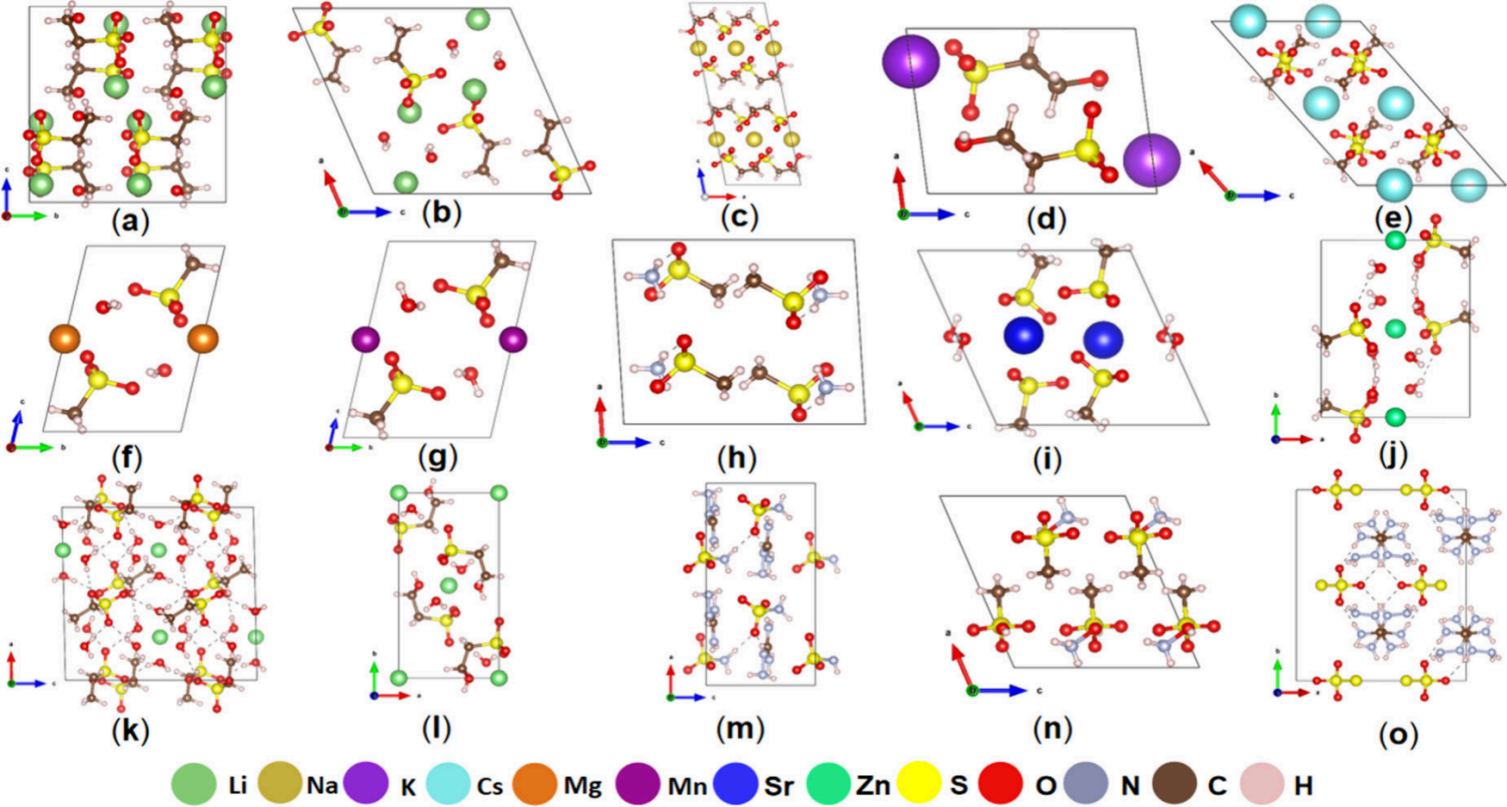
Overall structures of (a) LiSO_3_C_2_H_4_OH, (b) LiSO_3_C_2_H_5_·H_2_O, (c) NaSO_3_C_2_H_4_OH, (d) KSO_3_C_2_H_4_OH, (e) CsH(SO_3_CH_3_)_2_, (f) Mg(SO_3_CH_3_)_2_·2H_2_O, (g) Mn(SO_3_CH_3_)_2_·2H_2_O, (h) NH_4_SO_3_CH_3_, (i) Sr(SO_3_CH_3_)_2_·H_2_O, (j) Zn(SO_3_CH_3_)_2_·4H_2_O, (k) Zn(SO_3_C_2_H_4_OH)_2_·2H_2_O, (l) Zn(SO_3_C_2_H_5_)_2_·6H_2_O, (m) (CN_4_H_7_)SO_3_NH_2_, (n) (NH_3_OH)SO_3_CH_3_, and (o) (CN_4_H_7_)S_2_O_3_ compounds.

From Figure 3, the overall structural arrangement of all 15 compounds is shown. We can see the anion arrangement and understand how the anions and cations affect each other.^67^ For the anionic [SO_3_C_2_H_4_OH] groups, we find that as the cation moves from lithium to sodium to potassium ions, the angle between the anionic groups (based on the three oxygen atoms in the [SO_3_C_2_H_4_OH] groups) goes from 6.82° to 2.63° to 0°, which suggests that they are more aligned. For the [SO_3_CH_3_] group, Mg^2+^ and Mn^2+^ have similar ionic radii; therefore, the effect on the crystal space group and anion arrangement is limited, and only the cell parameters slightly change. Besides, [C(NH_2_)_3_], [NH_4_], [NH_3_OH], Cs^+^, and Sr^2+^ are chosen to obtain the wide bandgap. For the Zn^2+^ cation, Zn(SO_3_CH_3_)_2_·4H_2_O, Zn(SO_3_C_2_H_5_)_2_·6H_2_O, and Zn(SO_3_C_2_H_4_OH)_2_·2H_2_O were obtained to analyze the effect of these different anionic units on their optical properties. For the [SO_3_C_2_H_5_], [S_2_O_3_] and [SO_3_NH_2_] groups, LiSO_3_C_2_H_5_·H_2_O, (CN_4_H_7_)S_2_O_3_, and (CN_4_H_7_)SO_3_NH_2_ compounds were obtained to study optical properties, respectively.

### Characterization

**Figure 4:**
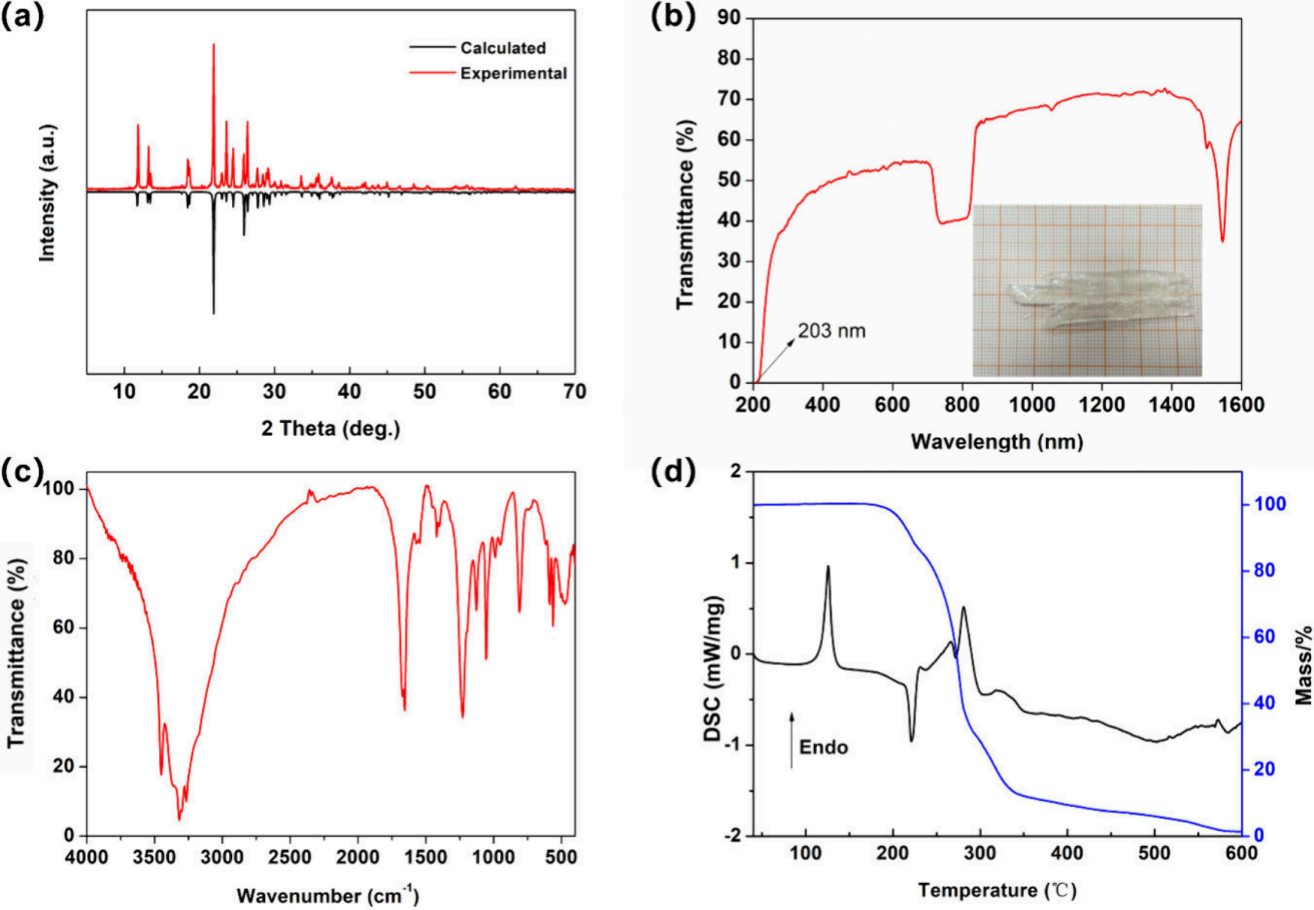
(a) Powder XRD patterns of (CN_4_H_7_)SO_3_NH_2_ crystals. (b) UV–vis–NIR transmittance spectrum and photograph of (CN_4_H_7_)SO_3_NH_2_ crystals (databar is 1 mm). (c) IR spectrum of (CN_4_H_7_)SO_3_NH_2_ crystals. (d) Thermogravimetric analysis–differential scanning calorimetry curves of (CN_4_H_7_)SO_3_NH_2_.

To make some initial assessments of the polycrystalline powder, the phase purities of LiSO_3_C_2_H_5_·H_2_O, NaSO_3_C_2_H_4_OH, KSO_3_C_2_H_4_OH, Mg(SO_3_CH_3_)_2_·2H_2_O, NH_4_SO_3_CH_3_, Sr(SO_3_CH_3_)_2_·H_2_O, Zn(SO_3_CH_3_)_2_·4H_2_O, and Zn(SO_3_C_2_H_4_OH)_2_·2H_2_O were confirmed by powder X-ray diffraction (Figure S1). We also performed UV–vis–NIR diffuse reflectance spectroscopy of their powders to obtain their cutoff edge, as shown in Figure S2. To obtain a more accurate bandgap, we grew some of the crystals and performed transmission spectroscopy tests as shown in Figure S3. After the transformation, the band gaps of the corresponding compounds were obtained and are summarized in Table 1. In the manuscript, we developed a detailed description of the characterization using the example of (CN_4_H_7_)SO_3_NH_2_. The powder XRD verified that the purity and intensity differences are mainly due to the preferred orientation in the preparation of the samples (Figure 4a). As shown in Figure 4b, millimeter-scale crystals were grown and polished slightly for transmission spectroscopy testing to more accurately define the cutoff edge. The cutoff edge is about 203 nm, corresponding to the band gap of 6.11 eV, and the appearance of an absorption peak at about 1550 nm can be specifically attributed to the appearance of [NH_2_] groups.

**Table 1:** Comparison of Optical Properties and Angles for the Compounds

formula	band gap (eV)	Δ*n* (@546 nm)	angle (deg)
NH_4_SO_3_CH_3_	6.89	0.030^exp^	0
Zn(SO_3_CH_3_)_2_·4H_2_O	6.81	0.046	13.766
Zn(SO_3_CH_2_CH_2_OH)_2_·2H_2_O	6.53	0.036	36.424
NaSO_3_CH_2_CH_2_OH	6.67	0.023	2.630
KSO_3_CH_2_CH_2_OH	6.49	0.024	0
Mg(SO_3_CH_3_)_2_·2H_2_O	6.67	0.025	0
Sr(SO_3_CH_3_)_2_·H_2_O	6.74		13.761
Zn(SO_3_C_2_H_5_)_2_·6H_2_O	6.53	0.036	56.847
LiSO_3_CH_2_CH_2_OH	7.71^a^	0.025	6.820
LiSO_3_C_2_H_5_·H_2_O	6.85	0.017	44.896
(NH_3_OH)SO_3_CH_3_	7.14^a^	0.043	45.411
CsHSO_3_CH_3_	7.50^a^	0.0066	89.942
(CN_4_H_7_)SO_3_NH_2_	6.11	0.155^exp^/0.153	/

a Calculated by HSE06.

According to the infrared spectrum of Figure 4c, the absorption peaks at 3453 and 3317 cm^–1^ can be attributed to the asymmetric and symmetric stretching vibrations of the [NH_2_] units. The peak near 1670 cm^–1^ can be attributed to the scissoring vibrations of the [NH_2_] units, and the twist of the [NH_2_] units appears at about 500 cm^–1^. The rocking vibration of the C–N bond can be observed at 1056 cm^–1^, which confirms the rationality of the [CN_4_H_7_] cation group. The antisymmetric and symmetric stretching and antisymmetric and symmetric deformation of the S–O bond can be observed at 1226, 1057, 586, and 563 cm^–1^, respectively. The vibration of the S–N bond appears at 810 cm^–1^, which shows the existence of the [SO_3_NH_2_] anion group. As shown in Figure 4d, the thermodynamic results reveal that (CN_4_H_7_)SO_3_NH_2_ could be stable up to 120 °C, and then it goes into total disintegration. The TG-DSC curves of the other compounds are shown in Figure S4, which show some underlying patterns by a sufficiently large number of results. In general, the water of crystallization will begin to be lost at about 100 °C, and the [SO_3_CH_3_], [SO_3_CH_2_CH_2_OH], and [SO_3_CH_2_CH_3_] groups will start to break down around 400, 200, and 350 °C, respectively.^68^

**Figure 5:**
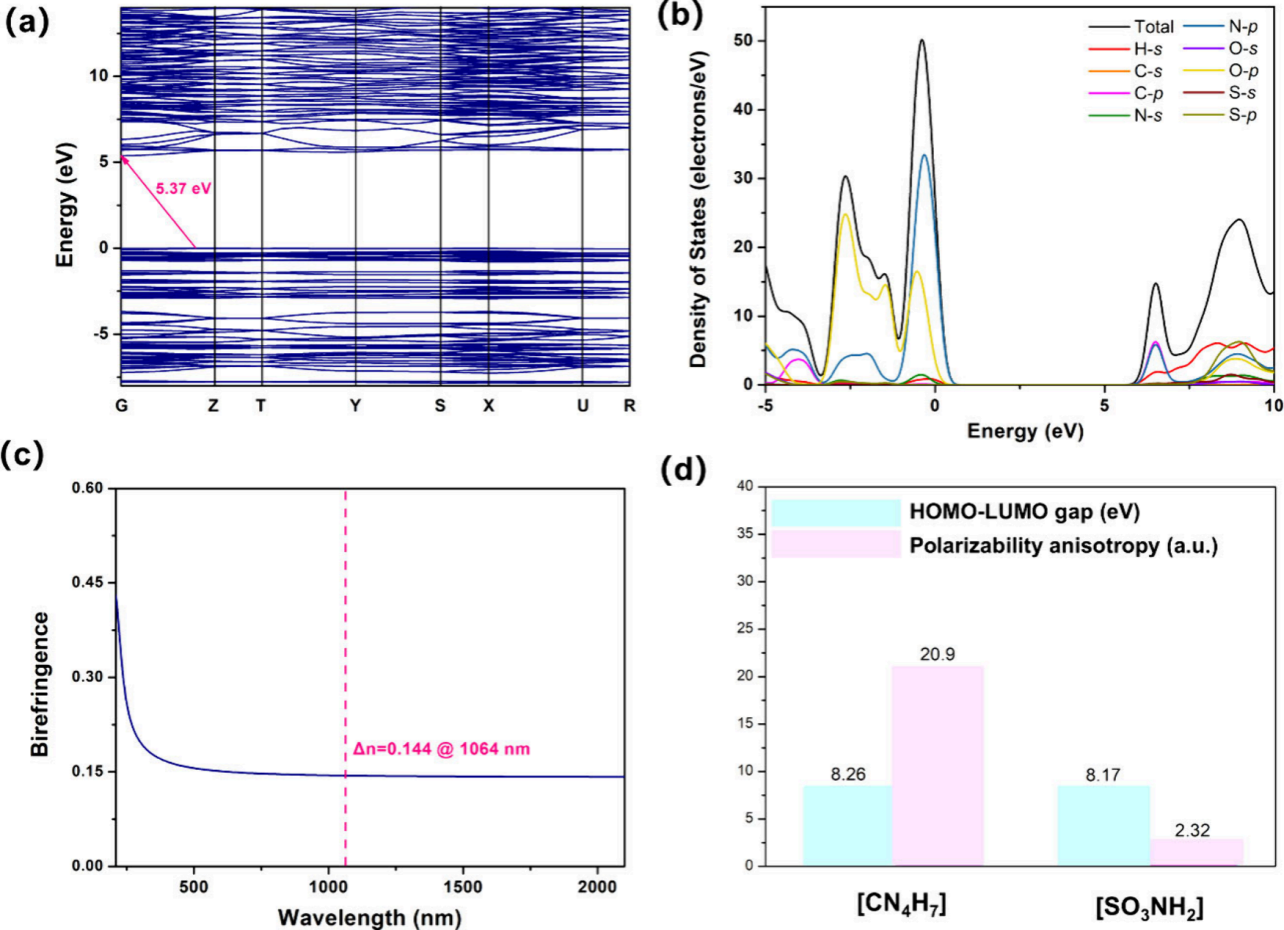
(a) Band structure. (b) Partial density of states. (c) Calculated birefringence at 1064 nm. (d) Optical properties of microscopic groups of (CN_4_H_7_)SO_3_NH_2_.

Based on first-principles, the relationship between optical properties and structure was analyzed within the CASTEP module with the plane-wave pseudopotential density functional theory (DFT) package, and specific parameter settings are shown in Table S5. To understand the relationship between structure and performance, the band structure, birefringence, total density of states (DOS), and partial density of states (PDOS) were computed by DFT (Figure 5). We considered the underestimation of the bandgap in the standard DFT calculations using generalized gradient approximation (GGA) due to discontinuities in the exchange-correlation energy.

To more precisely evaluate the optical properties, the difference between the experimental or calculated value by HSE06 and the calculated value by GGA functional is used as a scissor operator to obtain the optical properties, and the results are summarized and presented in Table 1. Taking (CN_4_H_7_)SO_3_NH_2_ as an example, the partial density of states (PDOS) with the orbitals near the Fermi level show that both the [CN_4_H_7_] and [SO_3_NH_2_] groups contribute to the enhanced birefringence. Like other compounds with a metal cation, the novel modification modules make a major contribution to optical properties (Figure S5). The main contribution of different units to birefringence is revealed using real-space atom-cutting (RSAC) methods, and the results show that the proportion of contribution to birefringence from [SO_3_NH_2_] reaches about 18% and the main contribution comes from the π-conjugated [CN_4_H_7_] units in Table S6.^72^ This verifies that the modified [SO_4_] units contribute positively to birefringence. Since it crystallizes in the noncentrosymmetric space group, its nonlinear coefficients are calculated, *d*_32_ = 0.29 pm/V, *d*_31_ = 0.21 pm/V, which is consistent with the experimental performance of about 0.1 × KDP.

**Figure 6:**
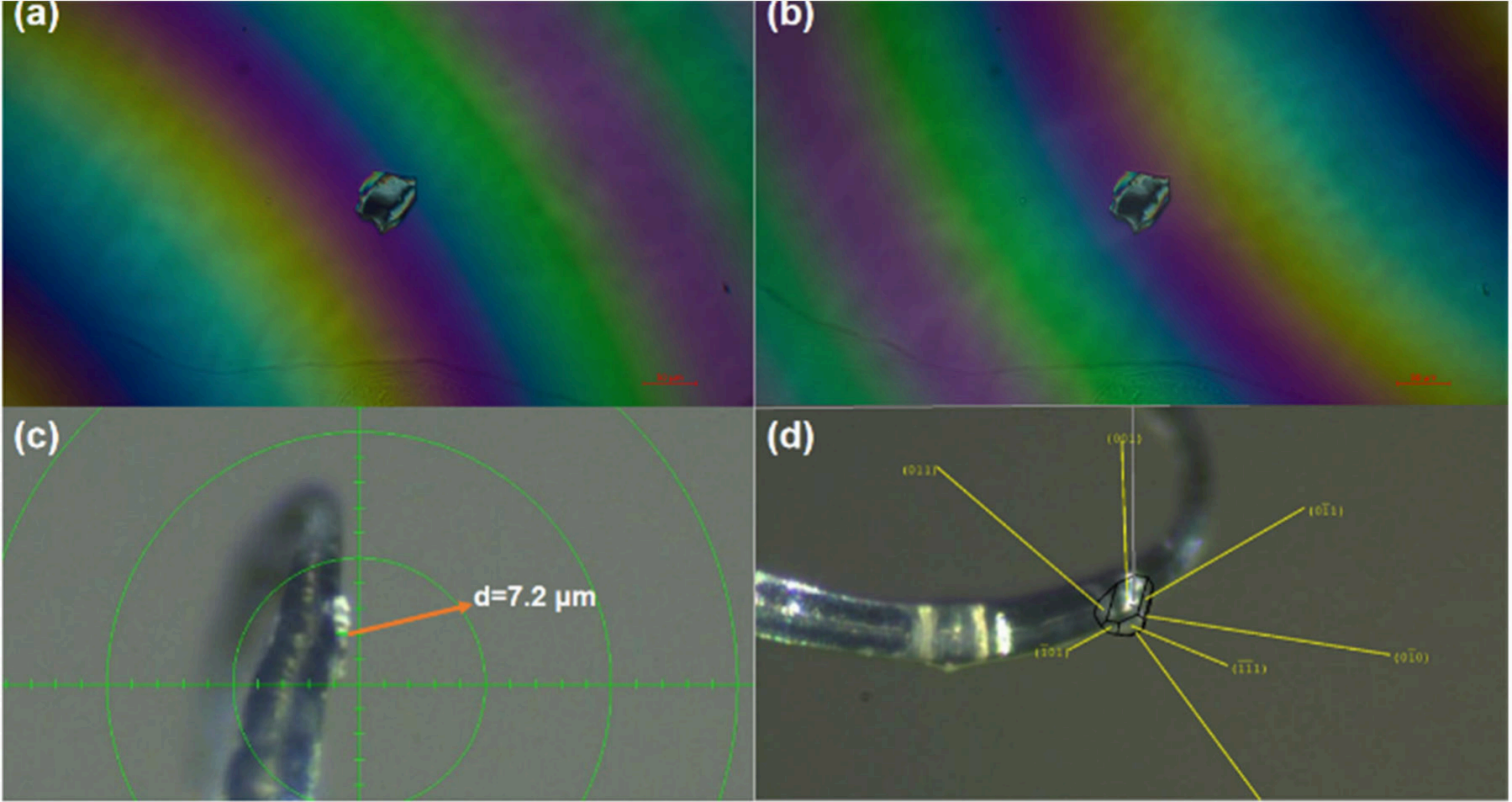
Birefringence test of (CN_4_H_7_)SO_3_NH_2_. (a) Positive rotation of compensation. (b) Negative rotation of compensation. (c) Thickness of the crystal. (d) Orientation of the selected crystal plane.

To verify the accuracy of our calculations about birefringence, we selected high-quality crystals with superior properties for evaluation. The birefringence of the (CN_4_H_7_)SO_3_NH_2_ wafer was tested with the polarizing microscope based on a (001) indexed crystal wafer. The retardation value of the (CN_4_H_7_)SO_3_NH_2_ crystal with a thickness of 7.2 μm is 1.116 μm by the Berek compensator specification. The result indicates that the birefringence of (CN_4_H_7_)SO_3_NH_2_ is about 0.155 at 546 nm (Figure 6), which is generally consistent with the theoretical calculation results (0.153@546 nm). By the same principle, a natural growth surface of NH_4_SO_3_CH_3_ with a thickness of 25.7 μm and a retardation value of 0.772 μm was selected and the birefringence was also experimentally measured to be greater than or equal to 0.030@546 nm (Figure S6).

Given the calculated birefringence by using the first-principles approach, the compounds LiSO_3_C_2_H_4_OH, LiSO_3_C_2_H_5_·H_2_O, NaSO_3_C_2_H_4_OH, KSO_3_C_2_H_4_OH, CsH(SO_3_CH_3_)_2_, (CN_4_H_7_)SO_3_NH_2_, Mg(SO_3_CH_3_)_2_·2H_2_O, NH_4_SO_3_CH_3_, Sr(SO_3_CH_3_)_2_·H_2_O, Zn(SO_3_CH_3_)_2_·4H_2_O, Zn(SO_3_C_2_H_4_OH)_2_·2H_2_O, (NH_3_OH)SO_3_CH_3_, and Zn(SO_3_C_2_H_5_)_2_·6H_2_O exhibit the phenomenon of hierarchy in birefringence, as shown in Table 1. In the context of (CN_4_H_7_)S_2_O_3_, it exhibits a birefringence of approximately 0.098 at 546 nm, with a bandgap of around 5.00 eV. This supports the previous assumption that the [S_2_O_3_] unit is not a suitable primitive in the short-wavelength region. As demonstrated in Figure 3, this shows that not all triangular bases of the [SO_3_-X] groups (X stands for ligand) are parallel, with varying dihedral angles (φ). The dihedral angles are listed in Table 1, which shows that the structures with smaller dihedral angles generally have larger birefringence. Additionally, the density of birefringence-active groups also impacts overall birefringence.

### Birefringence Analysis

**Figure 7:**
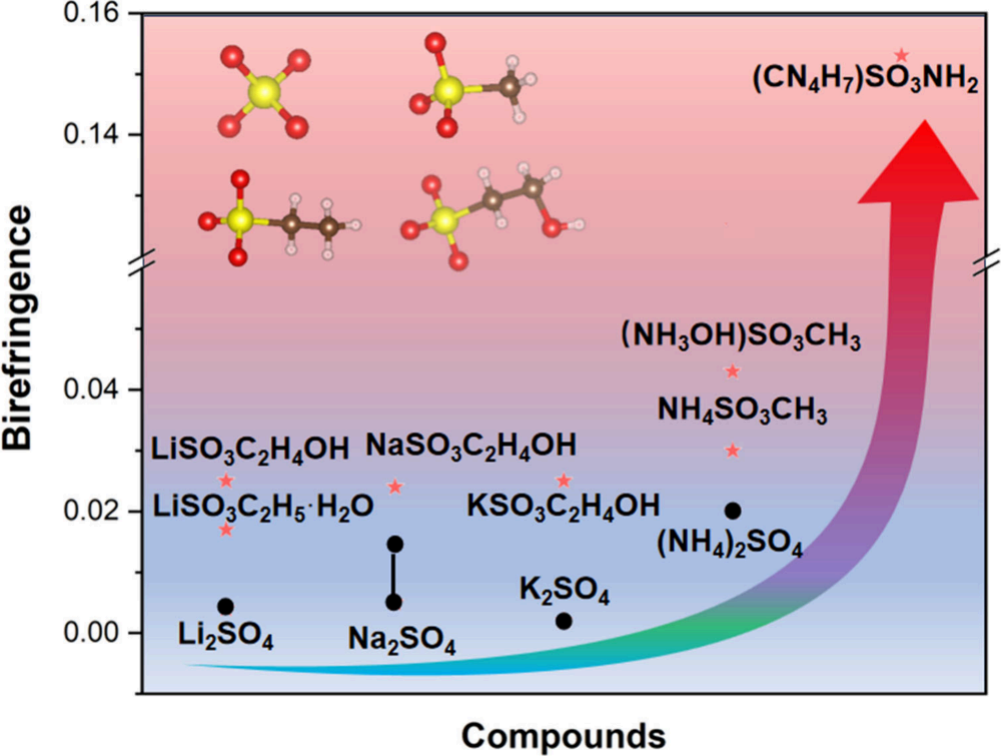
Comparison of birefringence for the designed and original compounds.

Li_2_SO_4_ behaves with a very small birefringence (0.004@546 nm), but LiSO_3_C_2_H_4_OH and LiSO_3_C_2_H_5_·H_2_O exhibit enhanced birefringence (0.025 and 0.017 @546 nm). A similar situation can be seen in Na_2_SO_4_ and K_2_SO_4_, and the birefringence also has been enhanced significantly. Furthermore, all of the compounds have short cutoff edges and possess relatively large birefringence through rational design, which can reach up to about 4–6 times the birefringence of the corresponding sulfate while maintaining a DUV band gap at the same time (Figure 7). Interestingly, (CN_4_H_7_)SO_3_NH_2_ behaves with about 35 times the birefringence of Li_2_SO_4_, which is a very significant order of magnitude improvement. These results confirm the analysis of the optical properties of microscopic primitives that the [SO_3_CH_2_CH_2_OH], and [SO_3_CH_2_CH_3_] groups by ligand substitution exhibit much larger polarizability anisotropy than that of the corresponding tetrahedral [SO_4_] group.

The same phenomena can be seen in Figure S7, and the birefringence of zinc-containing derivatives is improved in both.^69^ The anisotropy of the crystals is all improved by assembling modified modules and is also closely related to the alignment. If the angles of the effective primitives are distributed in a near-vertical arrangement, this is a very poor arrangement for birefringence, resulting in small birefringence, such as Li_2_RbLaB_18_O_30_ (Li_2_CsLaB_18_O_30_) with a birefringence of 0.003 (0.002) @532 nm.^70^ Since it is difficult to control the arrangement of the active moieties, the selection of the active moieties is very important. In this work, although CsHSO_3_CH_3_ (0.007@546 nm) with awful arrangement, still exhibits about triple the birefringence of Cs_2_SO_4_ (0.002@546 nm). As shown in Figure S8, the present work is compared with π-conjugated tailored sulfate crystals and commercial birefringent crystals.^71^ The results show that the crystals designed and synthesized in this work fill the gap in the wide-bandgap region, resulting in a balance between birefringence and bandgap.

Blindly exploring materials and then growing great crystals to assess birefringence can take a great deal of time. With advances in computational science, we obtain optical properties by calculating the overall structure of the crystals. Currently, the optical properties of crystals are mainly assessed from the macroscopic or overall structure of the crystal. To accelerate the exploration and evaluation of birefringent materials, we propose a new paradigm and try to start from the microscopic groups for rapid evaluation and synthesize compounds with target properties by molecular assembly.

Based on the new paradigm of ligand substitution for birefringence engineering, this paper focuses on addressing the small anisotropy of [SO_4_] groups. It begins with a microscopic approach to ligand substitution through crystal engineering, considering the microscopic HOMO–LUMO energy gap and anisotropy of the groups. We prioritize regions with high overall performance and HOMO–LUMO energy gap greater than 6.2 eV to increase the likelihood of achieving a balance between wide bandgap and anisotropy. To validate the approach, suitable cations were chosen, and the effectiveness of the selected birefringent active modules was confirmed in the short-wavelength region. The paper concludes by validating the chosen groups and the proposed strategy, which suggests a new paradigm for the future synthesis of novel short-wavelength birefringent crystals.

## Conclusion

In this work, we proposed and utilized a “ligand substitution” birefringence engineering approach, focusing on [SO_4_] modification, to fine-tune the optical anisotropy. This strategy leads to the discovery of new functional motifs composed of replacement atoms or modules, which significantly enhance optical anisotropy. We synthesized and calculated 15 compounds with selected functional modules, resulting in the discovery of nine new compounds. Theoretical studies from a microstructural perspective reveal a new generalized strategy in which the anisotropic electron distribution induced by new functional modules is responsible for enhanced birefringence. The derivatives can reach up to about 4–6 times the birefringence of the corresponding sulfate while still maintaining a wide band gap, which serves the purpose of our design and shows the validity of our proposed paradigm. This work provides a new paradigm for the enhancement of anisotropy and guides the future development of designing short-wavelength optical materials with great birefringence.

## Supporting Information Available

CIF and CheckCIF files for the crystal structures in this paper (ZIP)
PXRD patterns, TG and DSC curves, UV–vis–NIR diffuse reflectance or transmittance spectrum, electron band structure, calculated and experimental birefringent test, and PDOS curves (PDF)

## References

[ref1] Mutailipu M.; Poeppelmeier K. R.; Pan S. L. Borates: A Rich Source for Optical Materials. Chem. Rev. 2021, 121, 1130–1202. 10.1021/acs.chemrev.0c00796. 33307685

[ref2] Dodge M. J. Refractive properties of magnesium fluoride. Appl. Opt. 1984, 23, 1980–1985. 10.1364/AO.23.001980. 18212936

[ref3] Zhou G. Q.; Xu J.; Chen X. D.; Zhong H. Y.; Wang S. T.; Xu K.; Deng P. Z.; Gan F. X. Growth and Spectrum of a Novel Birefringent α-BaB_2_O_4_ Crystal. J. Cryst. Growth 1998, 191, 517–519. 10.1016/S0022-0248(98)00162-6.

[ref4] Sedlmeir F.; Zeltner R.; Leuchs G.; Schwefel H. G. L. High-Q MgF_2_ Whispering Gallery Mode Resonators for Refractometric Sensing in Aqueous Environment. Opt. Express 2014, 22, 30934–30942. 10.1364/OE.22.030934. 25607042

[ref5] Chen X. L.; Zhang B. B.; Zhang F. F.; Wang Y.; Zhang M.; Yang Z. H.; Poeppelmeier K. R.; Pan S. L. Designing a Deep-Ultraviolet Birefringent Material for Light Polarization. J. Am. Chem. Soc. 2018, 140, 16311–16319. 10.1021/jacs.8b10009. 30418021

[ref6] Liu T.; Shi W.; Tang W.; Liu Z.; Schroeder B. C.; Fenwick O.; Fuchter M. J. High Responsivity Circular Polarized Light Detectors Based on Quasi Two-Dimensional Chiral Perovskite Films. ACS Nano 2022, 16, 2682–2689. 10.1021/acsnano.1c09521. 35107990 PMC9007523

[ref7] Zhang F. F.; Chen X. L.; Zhang M.; Jin W. Q.; Han S. J.; Yang Z. H.; Pan S. L. An Excellent Deep Ultraviolet Birefringent Material Based on [BO_2_]_∞_ Infinite Chains. Light Sci. Appl. 2022, 11, 252 10.1038/s41377-022-00941-2. 35953466 PMC9372186

[ref8] Wu S. F.; Wang G. F.; Xie J. L.; Wu X. Q.; Zhang Y. F.; Lin X. Growth of Large Birefringent α-BBO Crystal. J. Cryst. Growth 2002, 245, 84–86. 10.1016/S0022-0248(02)01693-7.

[ref9] Mutailipu M.; Li F. M.; Jin C. C.; Yang Z. H.; Poeppelmeier K. R.; Pan S. L. Strong Nonlinearity Induced by Coaxial Alignment of Polar Chain and Dense [BO_3_] Units in CaZn_2_(BO_3_)_2_. Angew. Chem., Int. Ed. 2022, 61, e202202096 10.1002/anie.202202096. 35258151

[ref10] Gong P.; Liu X.; Kang L.; Lin Z. Inorganic Planar π-Conjugated Groups in Nonlinear Optical Crystals: Review and Outlook. Inorg. Chem. Front. 2020, 7, 839–852. 10.1039/C9QI01589B.

[ref11] Mutailipu M.; Zhang M.; Wu H. P.; Yang Z. H.; Shen Y. H.; Sun J. L.; Pan S. L. Ba_3_Mg_3_(BO_3_)_3_F_3_ Polymorphs with Reversible Phase Transition and High Performances as Ultraviolet Nonlinear Optical Materials. Nat. Commun. 2018, 9, 3089 10.1038/s41467-018-05575-w. 30082914 PMC6078997

[ref12] Zou G. H.; Ye N.; Huang L.; Lin X. S. Alkaline-alkaline Earth Fluoride Carbonate Crystals ABCO_3_F (A = K, Rb, Cs; B = Ca, Sr, Ba) as Nonlinear Optical Materials. J. Am. Chem. Soc. 2011, 133, 20001–20007. 10.1021/ja209276a. 22035561

[ref13] Shi G. Q.; Wang Y.; Zhang F. F.; Zhang B. B.; Yang Z. H.; Hou X. L.; Pan S. L.; Poeppelmeier K. R. Finding the Next Deep Ultraviolet Nonlinear Optical Material: NH_4_B_4_O_6_F. J. Am. Chem. Soc. 2017, 139, 10645–10648. 10.1021/jacs.7b05943. 28726399

[ref14] Choi M. H.; Li Y.; OK K. M. Designing Optical Anisotropy: Silver-Aminoalkylpyridine Nitrate Complexes with Tunable Structures. Inorg. Chem. 2024, 63, 2793–2802. 10.1021/acs.inorgchem.3c04328. 38258810

[ref15] Zhang Z. Z.; Wang Y.; Zhang B. B.; Yang Z. H.; Pan S. L. Polar Fluorooxoborate, NaB_4_O_6_F: a Promising Material for Ionic Conduction and Nonlinear Optics. Angew. Chem., Int. Ed. 2018, 57, 6577–6581. 10.1002/anie.201803392. 29663612

[ref16] Liu Y.; Shen Y.; Zhao S.; Luo J. Structure-property Relationship in Nonlinear Optical Materials with π-conjugated CO_3_ Triangles. Coord. Chem. Rev. 2020, 407, 213152 10.1016/j.ccr.2019.213152.

[ref17] Wang Y.; Zhang B. B.; Yang Z. H.; Pan S. L. Cation-Tuned Synthesis of Fluorooxoborates: Towards Optimal Deep-Ultraviolet Nonlinear Optical Materials. Angew. Chem., Int. Ed. 2018, 57, 2150–2154. 10.1002/anie.201712168. 29316132

[ref18] Peng G.; Lin C.; Ye N. NaZnCO_3_(OH): A High-Performance Carbonate Ultraviolet Nonlinear Optical Crystal Derived from KBe_2_BO_3_F_2_. J. Am. Chem. Soc. 2020, 142, 20542–20546. 10.1021/jacs.0c09866. 33237765

[ref19] Wang X. F.; Wang Y.; Zhang B. B.; Zhang F. F.; Yang Z. H.; Pan S. L. CsB_4_O_6_F: a Congruent-Melting Deep-Ultraviolet Nonlinear Optical Material by Combining Superior Functional Units. Angew. Chem., Int. Ed. 2017, 56, 14119–14123. 10.1002/anie.201708231. 28895656

[ref20] Zhou Y.; Guo Z. F.; Gu H. G.; Li Y. Q.; Song Y. P.; Liu S. Y.; Hong M. C.; Zhao S. G.; Luo J. H. A Solution-Processable Natural Crystal with Giant Optical Anisotropy for Efficient Manipulation of Light Polarization. Nat. Photonics 2024, 18, 922 10.1038/s41566-024-01461-8.

[ref21] Cheng M.; Jin W. Q.; Yang Z. H.; Pan S. L. Large Optical Anisotropy-Oriented Construction of Carbonate-Nitrate Chloride as Potential Ultraviolet Birefringent Materials. Chem. Sci. 2022, 13, 13482–13488. 10.1039/D2SC03771H. 36507155 PMC9685371

[ref22] Tudi A.; Han S. J.; Yang Z. H.; Pan S. L. Potential Optical Functional Crystals with Large Birefringence: Recent Advances and Future Prospects. Coord. Chem. Rev. 2022, 459, 214380 10.1016/j.ccr.2021.214380.

[ref23] Huang W. Q.; Zhao S. G.; Luo J. H. Recent Development of Non-π-Conjugated Deep Ultraviolet Nonlinear Optical Materials. Chem. Mater. 2022, 34, 5–28. 10.1021/acs.chemmater.1c02554.

[ref24] Shang Y.; Xu J.; Sha H.; Wang Z.; He C.; Su R.; Yang X.; Long X. Nonlinear Optical Inorganic Sulfates: The Improvement of the Phase Matching Ability Driven by the Structural Modulation. Coord. Chem. Rev. 2023, 494, 215345 10.1016/j.ccr.2023.215345.

[ref25] Song Y.; Yu H.; Li B.; Li X.; Zhou Y.; Li Y.; He C.; Zhang G.; Luo J.; Zhao S. A Ferroelectric Nonlinear Optical Crystal for Deep-UV Quasi-Phase-Matching. Adv. Funct. Mater. 2024, 34, 2310407 10.1002/adfm.202310407.

[ref26] Shao M. C.; Liang F.; Yu H. H.; Zhang H. J. Pushing Periodic-Disorder-Induced Phase Matching into the Deep-Ultraviolet Spectral Region: Theory and Demonstration. Light Sci. Appl. 2020, 9, 45 10.1038/s41377-020-0281-4. 32194959 PMC7078200

[ref27] Fan H.; Ye N.; Luo M. New Functional Groups Design toward High Performance Ultraviolet Nonlinear Optical Materials. Acc. Chem. Res. 2023, 56, 3099–3109. 10.1021/acs.accounts.3c00575. 37889615

[ref28] Hu C. H.; Shen C. J.; Zhou H.; Han J.; Yang Z. H.; Poeppelmeier K. R.; Zhang F.; Pan S. L. C_3_N_2_H_5_)B_3_O_3_F_2_(OH)_2_: Realizing Large Birefringence via a Synergistic Effect between Anion F/OH-ratio Optimization and Cation Activation. Small Struct. 2024, 5, 2400296 10.1002/sstr.202400296.

[ref29] Pan X.; Liu F.; Lin Z.; Kang L. Birefringent Dispersion Optimization to Achieve Superior Nonlinear Optical Phase Matching Deeper Solar-blind UV Band from KH_2_PO_4_ to BeH_3_PO_5_. Small 2024, 20, 2308811 10.1002/smll.202308811. 37988700

[ref30] Hu C. H.; Chu D. D.; Hou X. L.; Zhang F.; Han J. Exploration of Antimony(iii) Oxyhalides via Single-site Substitution in Quest of Large Birefringence. Inorg. Chem. Front. 2024, 11, 3367–3376. 10.1039/D4QI00564C.

[ref31] Bai Z. Y.; Ok K. M. Dramatically Improved Optical Anisotropy by Realizing Stereochemically Active Lone Pairs in a Sulfate System, K_2_SO_4_·HIO_3_. Inorg. Chem. Front. 2023, 10, 1919–1925. 10.1039/D3QI00192J.

[ref32] Cho S.; Ok K. M. LiRE(SO_4_)_2_ (RE = Y, Gd, Eu): Noncentrosymmetric Chiral Rare-earth Sulfates with Very Large Bandgaps. Mater. Chem. Front. 2022, 7, 65–71. 10.1039/D2QM00983H.

[ref33] Zhu P.; Gong P.; Wang Z.; Jiang H.; Zhao J.; Li C.; Lin Z.; Duan X.; Yu F. Enlargement of Bandgap and Birefringence in Nonlinear Optical Alkali-Metal Sulfate Crystals by the Substitution of Asymmetrical Non-π-Conjugated Cation. Adv. Opt. Mater. 2023, 11, 2301152 10.1002/adom.202301152.

[ref34] Li Y. Q.; Liang F.; Zhao S. G.; Li L. N.; Wu Z. Y.; Ding Q. R.; Liu S.; Lin Z. S.; Hong M. C.; Luo J. H. Two Non-π-Conjugated Deep-UV Nonlinear Optical Sulfates. J. Am. Chem. Soc. 2019, 141, 3833–3837. 10.1021/jacs.9b00138. 30791686

[ref35] Wang K.; Li X. F.; He C.; Li J. H.; An X. T.; Wei L.; Wei Q.; Wang G. M. NaSb_3_O_2_(SO_4_)_3_·H_2_O: A New Alkali-Metal Antimony-(III) Sulfate with a Unique Sb_6_O_20_H_4_ Unit and Moderate Birefringence. Cryst. Growth Des. 2022, 22, 478–484. 10.1021/acs.cgd.1c01096.

[ref36] Ge Y. W.; Wang Q.; Yang F.; Huang L.; Gao D. J.; Bi J.; Zou G. H. Tin Chloride Sulfates A_3_Sn_2_(SO_4_)_3-*x*_Cl_1+2*x*_ (A = K, Rb, Cs; *x* = 0, 1) as Multifunctional Optical Materials. Inorg. Chem. 2021, 60, 8322–8330. 10.1021/acs.inorgchem.1c01037. 33990136

[ref37] Dong X. H.; Huang L.; Zeng H. M.; Lin Z. E.; Ok K. M.; Zou G. H. High-Performance Sulfate Optical Materials Exhibiting Giant Second Harmonic Generation and Large Birefringence. Angew. Chem., Int. Ed. 2022, 61, e202116790 10.1002/anie.202116790. 34984782

[ref38] Lan Y.; Ren J.; Zhang P.; Dong X.; Huang L.; Cao L.; Gao D.; Zou G. ASb(SO_4_)_2_ (A = Rb, Cs): Two Short-Wave UV Antimony Sulfates Exhibiting Large Birefringence. Chin. Chem. Lett. 2024, 35, 108652 10.1016/j.cclet.2023.108652. 38265337

[ref39] Jiao D. X.; Zhang H. L.; Li X. F.; He C.; Li J. H.; Wei Q.; Yang G. Y. YSO_4_F·H_2_O: A Deep-Ultraviolet Birefringent Rare-Earth Sulfate Fluoride with Enhanced Birefringence Induced by Fluorinated Y-Centered Polyhedra. Inorg. Chem. 2023, 62, 17333–17340. 10.1021/acs.inorgchem.3c02632. 37823856

[ref40] Wu C.; Jiang X. X.; Hu Y. L.; Jiang C. B.; Wu T. H.; Lin Z. S.; Huang Z. P.; Humphrey M. G.; Zhang C. A Lanthanumammonium Sulfate Double Salt with a Strong SHG Response and Wide Deep-UV Transparency. Angew. Chem., Int. Ed. 2022, 134, e202115855 10.1002/ange.202115855. 34894166

[ref41] Hu C. H.; Cai X. T.; Wu M. F.; Yang Z. H.; Han J.; Pan S. L. Lone Pair-Driven Enhancement of Birefringence in Polar Alkali Metal Antimony Phosphates. Chem. Mater. 2022, 34, 4224–4231. 10.1021/acs.chemmater.2c00808.

[ref42] Wu C.; Wu T. H.; Jiang X. X.; Wang Z. J.; Sha H. Y.; Lin L.; Lin Z. S.; Huang Z. P.; Long X. F.; Humphrey M. G.; Zhang C. Large Second-Harmonic Response and Giant Birefringence of CeF_2_(SO_4_) Induced by Highly Polarizable Polyhedra. J. Am. Chem. Soc. 2021, 143, 4138–4142. 10.1021/jacs.1c00416. 33625206

[ref43] Han Y.; Zhao X.; Xu F.; Li B.; Ye N.; Luo M. HgSO_4_: An Excellent Mid-Infrared Sulfate Nonlinear Optical Crystal with Wide Band Gap and Strong Second Harmonic Generation Response. J. Alloys Compd. 2022, 902, 163727 10.1016/j.jallcom.2022.163727.

[ref44] Liang M. L.; Zhang Y. J.; Izvarin E.; Waters M. J.; Rondinelli J. M.; Halasyamani P. S. Metal Methanesulfonates with Mixed Anionic Groups with Large Band Gaps and Enhanced Birefringence. Chem. Mater. 2024, 36, 2113–2123. 10.1021/acs.chemmater.3c03278.

[ref45] Sha H. Y.; Xu J. X.; Huang L. X.; Xiong Z. Y.; Wang Z. J.; Su R. B.; He C.; Yang X. M.; Long X. F. Alkali Metal Sulfate: a New Non-π-Conjugated Deep-Ultraviolet Quasi-Phase Matching Crystal. Scr. Mater. 2022, 217, 114764 10.1016/j.scriptamat.2022.114764.

[ref46] Hu C. H.; Wu M. F.; Zhang M.; Han J.; Hou X. L.; Zhang F.; Yang Z. H.; Pan S. L. Cation Activation”: An Effective Strategy for the Enhancement of Birefringence. Adv. Opt. Mater. 2023, 11, 2300579 10.1002/adom.202300579.

[ref47] Xiong L.; Wu L. M.; Chen L. A General Principle for DUV NLO Materials: π-conjugated Confinement Enlarges Band Gap. Angew. Chem., Int. Ed. 2021, 60, 25063–25067. 10.1002/anie.202110740. 34532933

[ref48] Mutailipu M.; Han J.; Li Z.; Li F. M.; Li J. J.; Zhang F. F.; Long X. F.; Yang Z. H.; Pan S. L. Achieving the Full-wavelength Phase-matching for Efficient Nonlinear Optical Frequency Conversionin C(NH_2_)_3_BF_4_. Nat. Photonics 2023, 17, 694–701. 10.1038/s41566-023-01228-7.

[ref49] Jin W. Q.; Zhang W. Y.; Tudi A.; Wang L. Y.; Zhou X.; Yang Z. H.; Pan S. L. Fluorine-Driven Enhancement of Birefringence in the Fluorooxosulfate: A Deep Evaluation from a Joint Experimental and Computational Study. Adv. Sci. 2021, 8, 2003594 10.1002/advs.202003594. PMC8336506 34085784

[ref50] Xia M.; Li F. M.; Mutailipu M.; Han S. J.; Yang Z. H.; Pan S. L. Discovery of First Magnesium Fluorooxoborate with Stable Fluorine Terminated Framework for Deep-UV Nonlinear Optical Application. Angew. Chem., Int. Ed. 2021, 60, 14650–14656. 10.1002/anie.202103657. 33871912

[ref51] Zhang Z. Z.; Wang Y.; Zhang B. B.; Yang Z. H.; Pan S. L. CaB_5_O_7_F_3_: A Beryllium-Free Alkaline-Earth Fluorooxoborate Exhibiting Excellent Nonlinear Optical Performances. Inorg. Chem. 2018, 57, 4820–4823. 10.1021/acs.inorgchem.8b00531. 29663805

[ref52] Mutailipu M.; Zhang M.; Zhang B. B.; Wang L. Y.; Yang Z. H.; Zhou X.; Pan S. L. SrB_5_O_7_F_3_ Functionalized with [B_5_O_9_F_3_]^6–^ Chromophores: Accelerating the Rational Design of Deep-Ultraviolet Nonlinear Optical Materials. Angew. Chem., Int. Ed. 2018, 57, 6095–6099. 10.1002/anie.201802058. 29498468

[ref53] Luo M.; Liang F.; Song Y. X.; Zhao D.; Xu F.; Ye N.; Lin Z. S. M2B10O14F6 (M = Ca, Sr): Two Noncentrosymmetric Alkaline-Earth Fluorooxoboratesas Promising Next-Generation Deep-ultraviolet Nonlinear Optical Materials. J. Am. Chem. Soc. 2018, 140, 3884–3887. 10.1021/jacs.8b01263. 29517902

[ref54] Liu K. T.; Han J.; Baiheti T.; Li F. M.; Wei Z. L.; Yang Z. H.; Mutailipu M.; Pan S. L. Finding a Series of BaBOF_3_ Fluorooxoborate Polymorphs with Tunable Symmetries: A Simple but Flexible Case. Chem. Mater. 2021, 33, 7905–7913. 10.1021/acs.chemmater.1c03020.

[ref55] Hu C. H.; Wu M. F.; Han J.; Yang Z. H.; Han S. J.; Pan S. L. New antimony Fluorooxoborates with Strong Birefringence and Unprecedented Structural Characterisation. Chem. Commun. 2024, 60, 2653–2656. 10.1039/D3CC05784D. 38348788

[ref56] Zhang B. B.; Shi G. Q.; Yang Z. H.; Zhang F. F.; Pan S. L. Fluorooxoborates: Beryllium-free Deep-ultraviolet Nonlinear Optical Materials without Layered Growth. Angew. Chem., Int. Ed. 2017, 56, 3916–3919. 10.1002/anie.201700540. 28251767

[ref57] Li F. M.; Jin W. Q.; An R.; Mutailipu M.; Pan S. L.; Yang Z. H. Covalently Bonded Fluorine Optimizing Deep-ultraviolet Nonlinear Optical Performance of Fluorooxoborates. Sci. Bull. 2024, 69, 1192–1196. 10.1016/j.scib.2024.03.007. 38503652

[ref58] Qiu H.; Li F.; Jin C.; Yang Z.; Li J.; Pan S.; Mutailipu M. Fluorination Strategy Towards Symmetry Breaking of Boron-centered Tetrahedron for Polyfluorinated Optical Crystals. Angew. Chem., Int. Ed. 2024, 63, e202316194 10.1002/anie.202316194. 38009443

[ref59] Hu C. H.; Li H. M.; Han J.; Hou X. L.; Yang Z. H.; Pan S. L. NaB(OH)_3_CH_3_: a Deep-ultraviolet Optical Crystal with Unprecedented Methyl-modified [B(OH)_3_CH_3_] Units. J. Mater. Chem. C 2024, 12, 7916–7920. 10.1039/D4TC01288G.

[ref60] Tian H.; Lin C.; Zhao X.; Xu F.; Wang C.; Ye N.; Luo M. Ba(SO_3_CH_3_)_2_: a Deep-ultraviolet Transparent Crystal with Excellent Optical Nonlinearity Based on a New Polar Non-π-Conjugated NLO Building Unit SO_3_CH_3_. CCS Chem. 2023, 5, 2497–2505. 10.31635/ccschem.023.202202582.

[ref61] Shang Y. R.; Sha H. Y.; Wang Z. J.; Su R. B.; He C.; Yang X. M.; Long X. F. The Birefringence Modulation in Short-wave Ultraviolet Sulfates with Functional π-conjugated Cations and Polymerized Heteroleptic Tetrahedral Anions. Adv. Opt. Mater. 2024, 12, 2302844 10.1002/adom.202302844.

[ref62] Wang X. F.; Leng X. D.; Kuk Y.; Lee J.; Jing Q.; Ok K. M. Deep-ultraviolet Transparent Mixed Metal Sulfamates with Enhanced Nonlinear Optical Properties and Birefringence. Angew. Chem., Int. Ed. 2024, 63, e2023154 10.1002/anie.202315434. 37973618

[ref63] Wang X. F.; Li Y.; Chen Z. L.; Lee J.; Zhang F. F.; Poeppelmeier K. R.; Pan S. L.; Ok K. M. Sr(NO_3_)(NH_2_SO_3_)·H_2_O: First Nitrate Sulfamate Revealing Remarkable Second Harmonic Generation and Optimized Birefringence. Small Struct. 2023, 4, 2300274 10.1002/sstr.202300274.

[ref64] Tian H. T.; Ye N.; Luo M. Sulfamide: a Promising Deep-ultraviolet Nonlinear Optical Crystal Assembled from Polar Covalent [SO_2_(NH_2_)_2_] Tetrahedra. Angew. Chem., Int. Ed. 2022, 61, e202200395 10.1002/anie.202200395. 35179290

[ref65] Hao X.; Luo M.; Lin C. S.; Peng G.; Xu F.; Ye N. M(NH_2_SO_3_)_2_ (M = Sr, Ba): Two Deep-Ultraviolet Transparent Sulfamates Exhibiting Strong Second Harmonic Generation Responses and Moderate Birefringence. Angew. Chem., Int. Ed. 2021, 60, 7621–7625. 10.1002/anie.202016372. 33470036

[ref66] Xu B.; Gong P.; Liu F.; Zhang X.; Huo H.; Lin Z. (SO_3_CF_3_)^−^: A Non-π-Conjugated Motif for Nonlinear Optical Crystals Transparent into the Deep-Ultraviolet Region. Adv. Opt. Mater. 2024, 12, 2301725 10.1002/adom.202301725.

[ref67] Lu J.; Lian Y. K.; Xiong L.; Wu Q. R.; Zhao M.; Shi K. X.; Chen L.; Wu L. M. How to Maximize Birefringence and Nonlinearity of π-conjugated Cyanurates. J. Am. Chem. Soc. 2019, 141, 16151–16159. 10.1021/jacs.9b08851. 31513386

[ref68] Wang M.; Song Z. G.; Jiang H.; Gong H. Thermal Decomposition of Metal Methanesulfonates in Air. J. Therm. Anal. Calorim. 2009, 98, 801–806. 10.1007/s10973-009-0119-z.

[ref69] Zhou Y.; Zhang X. Y.; Xiong Z. Y.; Long X. F.; Li Y. Q.; Chen Y. X.; Chen X.; Zhao S. G.; Lin Z. S.; Luo J. H. Non-π-Conjugated Deep-Ultraviolet Nonlinear Optical Crystal K_2_Zn_3_(SO_4_)(HSO_4_)_2_F_4_. J. Phys. Chem. Lett. 2021, 12, 8280–8284. 10.1021/acs.jpclett.1c01533. 34425677

[ref70] Zhang Z. Y.; Xu D.; Abudukadi T.; Yang Z. H.; Han S. J.; Pan S. L. Design of Deep-ultraviolet Zero-order Waveplate Materials Using LiB_3_O_5_ as the Template. Sci. China Mater. 2024, 67, 914–920. 10.1007/s40843-023-2743-5.

[ref71] Bai Z. Y.; Ok K. M. Designing Sulfate Crystals with Strong Optical Anisotropy through π-Conjugated Tailoring. Angew. Chem., Int. Ed. 2024, 63, e202315311 10.1002/anie.202315311. 37888616

[ref72] Lin J.; Lee M. H.; Liu Z. P.; Chen C. T.; Pickard C. J. Mechanism for Linear and Nonlinear Optical Effects in β-BaB_2_O_4_ Crystals. Phys. Rev. B 1999, 60, 13380–13389. 10.1103/PhysRevB.60.13380.

