# Supplementary material for: The New Paradigm of Ligand Substitution-Driven Enhancement of Anisotropy from SO4 Units in Short-Wavelength Region
Source: ACS Cent Sci. 2024 Nov 27;10(12):2312–20. doi: 10.1021/acscentsci.4c01401 (PMC11673188; doi:10.1021/acscentsci.4c01401)

checkCIF/PLATON report

You have not supplied any structure factors. As a result the full set of tests cannot be run.

THIS REPORT IS FOR GUIDANCE ONLY. IF USED AS PART OF A REVIEW PROCEDURE FOR PUBLICATION, IT SHOULD NOT REPLACE THE EXPERTISE OF AN EXPERIENCED CRYSTALLOGRAPHIC REFEREE.

No syntax errors found. CIF dictionary Interpreting this report

Datablock: 6788

```
Bond precision:   S- C = 0.0040 A          Wavelength=0.71073

Cell:             a=5.157(3)         b=5.687(3)        c=8.545(5)
                  alpha=77.91(2)     beta=74.92(2)     gamma=89.03(2)
Temperature:      273 K

                 Calculated                  Reported
Volume           236.4(2)                    236.5(2)
Space group      P -1                        P -1
Hall group       -P 1                        -P 1
Moiety formula   C2 H10 Mn O8 S2             C2 H10 Mn O8 S2
Sum formula      C2 H10 Mn O8 S2             C2 H10 Mn O8 S2
Mr               281.16                      281.16
Dx,g cm-3        1.975                       1.975
Z                1                           1
Mu (mm-1)        1.850                       1.850
F000             143.0                       143.0
F000'            143.67
h,k,lmax         6,7,11                      6,7,11
Nref             1096                        1085
Tmin,Tmax        0.798,0.850                 0.557,0.746
Tmin'            0.798

Correction method= # Reported T Limits: Tmin=0.557 Tmax=0.746
AbsCorr = MULTI-SCAN

Data completeness= 0.990          Theta(max)= 27.562

                                                      wR2(reflections)=
R(reflections)= 0.0366( 948)                          0.0967( 1085)

S = 1.082                  Npar= 67
```

The following ALERTS were generated. Each ALERT has the format
test-name_ALERT_alert-type_alert-level.
Click on the hyperlinks for more details of the test.

Alert level C

```
PLAT094_ALERT_2_C Ratio of Maximum / Minimum Residual Density ....       2.86 Report
PLAT242_ALERT_2_C Low    'MainMol' Ueq as Compared to Neighbors of        Mn1 Check
PLAT242_ALERT_2_C Low    'MainMol' Ueq as Compared to Neighbors of         S1 Check
```

Alert level G

```
PLAT004_ALERT_5_G Polymeric Structure Found with Maximum Dimension          1 Info
PLAT007_ALERT_5_G Number of Unrefined Donor-H Atoms .............           1 Report
              H2
PLAT154_ALERT_1_G The s.u.'s on the Cell Angles are Equal ..(Note)       0.02 Degree
PLAT199_ALERT_1_G Reported _cell_measurement_temperature ..... (K)        273 Check
PLAT200_ALERT_1_G Reported   _diffrn_ambient_temperature ..... (K)        273 Check
PLAT794_ALERT_5_G Tentative Bond Valency for Mn1       (II)      .       2.16 Info
```

0 **ALERT level A** = Most likely a serious problem - resolve or explain
0 **ALERT level B** = A potentially serious problem, consider carefully
3 **ALERT level C** = Check. Ensure it is not caused by an omission or oversight
6 **ALERT level G** = General information/check it is not something unexpected

3 ALERT type 1 CIF construction/syntax error, inconsistent or missing data
3 ALERT type 2 Indicator that the structure model may be wrong or deficient
0 ALERT type 3 Indicator that the structure quality may be low
0 ALERT type 4 Improvement, methodology, query or suggestion
3 ALERT type 5 Informative message, check

It is advisable to attempt to resolve as many as possible of the alerts in all categories. Often the minor alerts point to easily fixed oversights, errors and omissions in your CIF or refinement strategy, so attention to these fine details can be worthwhile. In order to resolve some of the more serious problems it may be necessary to carry out additional measurements or structure refinements. However, the purpose of your study may justify the reported deviations and the more serious of these should normally be commented upon in the discussion or experimental section of a paper or in the "special_details" fields of the CIF. checkCIF was carefully designed to identify outliers and unusual parameters, but every test has its limitations and alerts that are not important in a particular case may appear. Conversely, the absence of alerts does not guarantee there are no aspects of the results needing attention. It is up to the individual to critically assess their own results and, if necessary, seek expert advice.

Publication of your CIF in IUCr journals

A basic structural check has been run on your CIF. These basic checks will be run on all CIFs submitted for publication in IUCr journals (*Acta Crystallographica*, *Journal of Applied Crystallography*, *Journal of Synchrotron Radiation*); however, if you intend to submit to *Acta Crystallographica Section C* or *E* or *IUCrData*, you should make sure that full publication checks are run on the final version of your CIF prior to submission.

Publication of your CIF in other journals

Please refer to the *Notes for Authors* of the relevant journal for any special instructions relating to CIF submission.

PLATON version of 15/07/2024; check.def file version of 15/07/2024

Datablock 6788 - ellipsoid plot

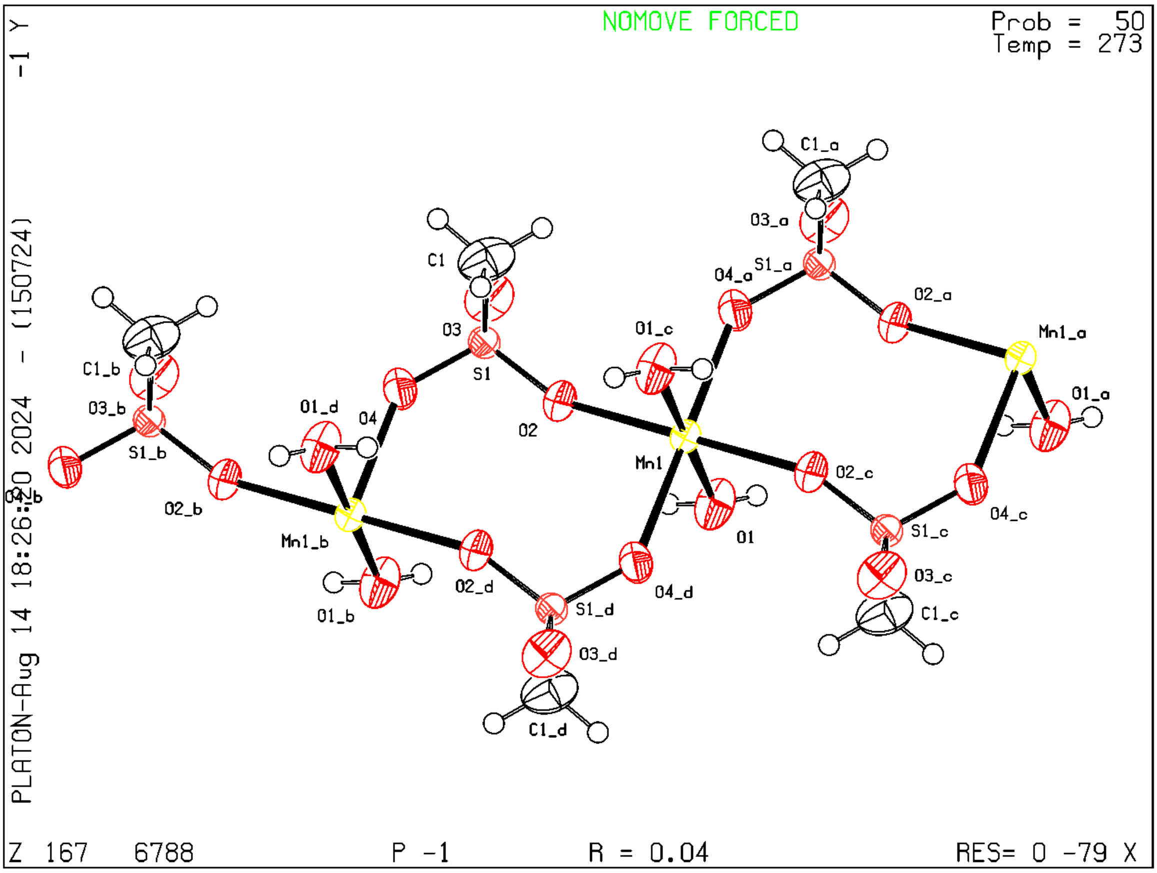

Supplement: Supplementary file 1 — oc4c01401_si_001.zip [file oc4c01401_si_001.zip › MnS2C2O8H10_checkcif.pdf]
