# Supplementary material for: The New Paradigm of Ligand Substitution-Driven Enhancement of Anisotropy from SO4 Units in Short-Wavelength Region
Source: ACS Cent Sci. 2024 Nov 27;10(12):2312–20. doi: 10.1021/acscentsci.4c01401 (PMC11673188; doi:10.1021/acscentsci.4c01401)

checkCIF/PLATON report

You have not supplied any structure factors. As a result the full set of tests cannot be run.

THIS REPORT IS FOR GUIDANCE ONLY. IF USED AS PART OF A REVIEW PROCEDURE FOR PUBLICATION, IT SHOULD NOT REPLACE THE EXPERTISE OF AN EXPERIENCED CRYSTALLOGRAPHIC REFEREE.

No syntax errors found.   CIF dictionary   Interpreting this report

Datablock: 2

Bond precision: S- C = 0.0040 A   Wavelength=0.71073

Cell:	a=7.4927(7)	b=7.2826(7)	c=9.2278(9)
	alpha=90	beta=93.372(5)	gamma=90
Temperature:	273 K		

	Calculated	Reported
Volume	502.66(8)	502.66(8)
Space group	C 2/m	C 1 2/m 1
Hall group	-C 2y	-C 2y
Moiety formula	C H3 O3 S, H4 N	C H3 O3 S, 0.5(H8 N2)
Sum formula	C H7 N O3 S	C H7 N O3 S
Mr	113.14	113.14
Dx,g cm-3	1.495	1.495
Z	4	4
Mu (mm-1)	0.529	0.529
F000	240.0	240.0
F000’	240.63	
h,k,lmax	9,9,11	9,9,11
Nref	621	622
Tmin,Tmax	0.856,0.941	0.663,0.746
Tmin’	0.835	

Correction method= # Reported T Limits: Tmin=0.663 Tmax=0.746
AbsCorr = MULTI-SCAN

Data completeness= 1.002   Theta(max)= 27.516

R(reflections)= 0.0438(591)   wR2(reflections)= 0.1314(622)

S = 1.164   Npar= 35

The following ALERTS were generated. Each ALERT has the format
test-name_ALERT_alert-type_alert-level.
Click on the hyperlinks for more details of the test.

Alert level C

```
PLAT042_ALERT_1_C Calc. and Reported MoietyFormula Strings  Differ     Please Check
              Calc: C H3 O3 S, H4 N
              Rep.: C H3 O3 S, 0.5(H8 N2)
PLAT242_ALERT_2_C Low    'MainMol' Ueq as Compared to Neighbors of          S1 Check
```

Alert level G

```
PLAT007_ALERT_5_G Number of Unrefined Donor-H Atoms .............           4 Report
              H4    H5    H6    H7
PLAT199_ALERT_1_G Reported _cell_measurement_temperature ..... (K)        273 Check
PLAT200_ALERT_1_G Reported   _diffrn_ambient_temperature ..... (K)        273 Check
PLAT299_ALERT_4_G Atom Site Occupancy Constrained at ............         0.5 Check
              H1      H2      H3      H4      H5
PLAT304_ALERT_4_G Non-Integer Number of Atoms in ..... (Resd    2)       2.50 Check
PLAT789_ALERT_4_G Atoms with Negative _atom_site_disorder_group  #          3 Check
PLAT822_ALERT_4_G CIF-embedded .res Contains Negative PART Numbers          1 Check
```

0 **ALERT level A** = Most likely a serious problem - resolve or explain
0 **ALERT level B** = A potentially serious problem, consider carefully
2 **ALERT level C** = Check. Ensure it is not caused by an omission or oversight
7 **ALERT level G** = General information/check it is not something unexpected

3 ALERT type 1 CIF construction/syntax error, inconsistent or missing data
1 ALERT type 2 Indicator that the structure model may be wrong or deficient
0 ALERT type 3 Indicator that the structure quality may be low
4 ALERT type 4 Improvement, methodology, query or suggestion
1 ALERT type 5 Informative message, check

It is advisable to attempt to resolve as many as possible of the alerts in all categories. Often the minor alerts point to easily fixed oversights, errors and omissions in your CIF or refinement strategy, so attention to these fine details can be worthwhile. In order to resolve some of the more serious problems it may be necessary to carry out additional measurements or structure refinements. However, the purpose of your study may justify the reported deviations and the more serious of these should normally be commented upon in the discussion or experimental section of a paper or in the "special_details" fields of the CIF. checkCIF was carefully designed to identify outliers and unusual parameters, but every test has its limitations and alerts that are not important in a particular case may appear. Conversely, the absence of alerts does not guarantee there are no aspects of the results needing attention. It is up to the individual to critically assess their own results and, if necessary, seek expert advice.

Publication of your CIF in IUCr journals

A basic structural check has been run on your CIF. These basic checks will be run on all CIFs submitted for publication in IUCr journals (*Acta Crystallographica*, *Journal of Applied Crystallography*, *Journal of Synchrotron Radiation*); however, if you intend to submit to *Acta Crystallographica Section C* or *E* or *IUCrData*, you should make sure that full publication checks are run on the final version of your CIF prior to submission.

Publication of your CIF in other journals

Please refer to the *Notes for Authors* of the relevant journal for any special instructions relating to CIF submission.

PLATON version of 15/07/2024; check.def file version of 15/07/2024

Datablock 2 - ellipsoid plot

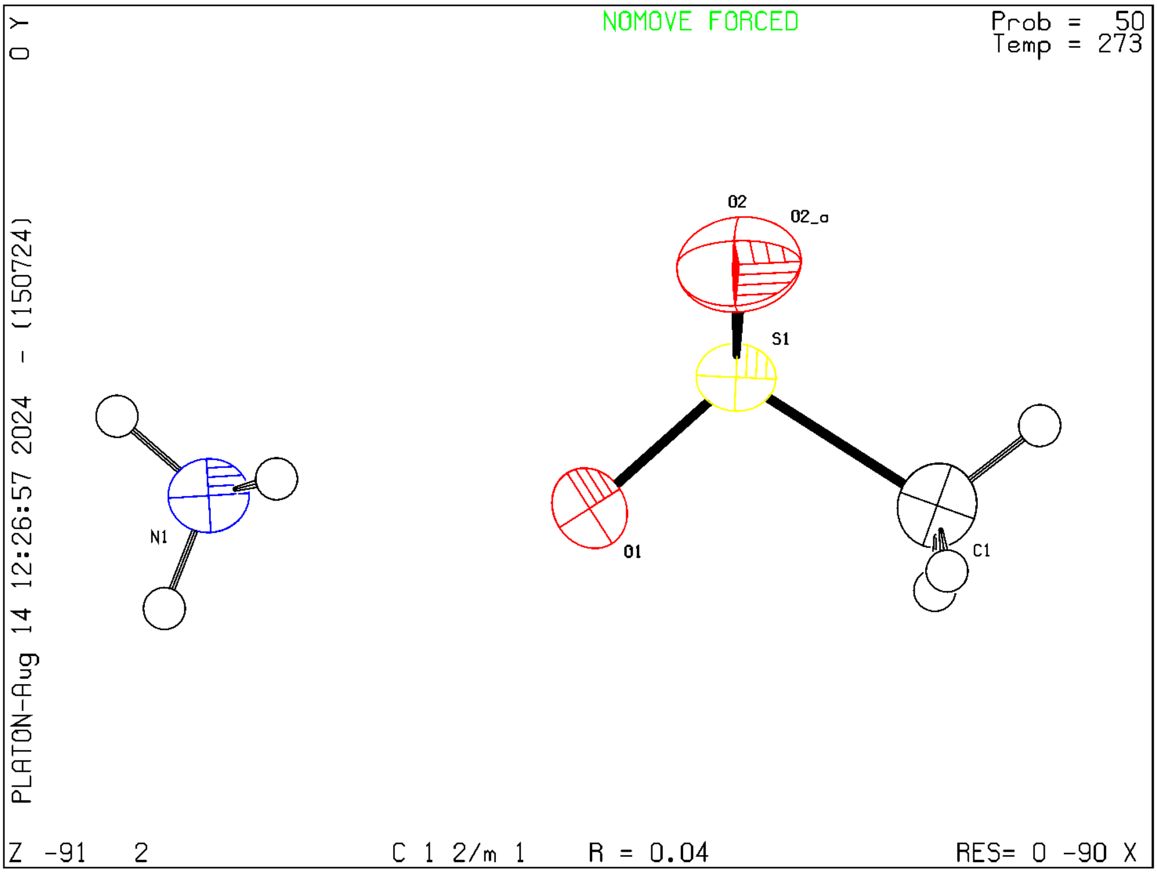

Supplement: Supplementary file 1 — oc4c01401_si_001.zip [file oc4c01401_si_001.zip › NH4SO3CH3_checkcif.pdf]
