# Supplementary material for: The New Paradigm of Ligand Substitution-Driven Enhancement of Anisotropy from SO4 Units in Short-Wavelength Region
Source: ACS Cent Sci. 2024 Nov 27;10(12):2312–20. doi: 10.1021/acscentsci.4c01401 (PMC11673188; doi:10.1021/acscentsci.4c01401)

checkCIF/PLATON report

You have not supplied any structure factors. As a result the full set of tests cannot be run.

THIS REPORT IS FOR GUIDANCE ONLY. IF USED AS PART OF A REVIEW PROCEDURE FOR PUBLICATION, IT SHOULD NOT REPLACE THE EXPERTISE OF AN EXPERIENCED CRYSTALLOGRAPHIC REFEREE.

No syntax errors found. CIF dictionary Interpreting this report

Datablock: 6

Bond precision: S- C = 0.0040 A Wavelength=0.71073

Cell:	a=8.6144(9)	b=6.0625(6)	c=9.0776(11)
	alpha=90	beta=113.126(4)	gamma=90
Temperature:	273 K		

	Calculated	Reported
Volume	435.98(8)	435.98(8)
Space group	P 21/m	P 1 21/m 1
Hall group	-P 2yb	-P 2yb
Moiety formula	C2 H8 O7 S2 Sr	C2 H8 O7 S2 Sr
Sum formula	C2 H8 O7 S2 Sr	C2 H8 O7 S2 Sr
Mr	295.82	295.82
Dx,g cm-3	2.253	2.253
Z	2	2
Mu (mm-1)	6.661	6.661
F000	292.0	292.0
F000'	289.64	
h,k,lmax	11,7,11	11,7,11
Nref	1097	1098
Tmin,Tmax	0.404,0.487	0.411,0.746
Tmin'	0.373	

Correction method= # Reported T Limits: Tmin=0.411 Tmax=0.746
AbsCorr = MULTI-SCAN

Data completeness= 1.001 Theta(max)= 27.531

R(reflections)= 0.0203(1038) wR2(reflections)= 0.0486(1098)

S = 1.171 Npar= 71

The following ALERTS were generated. Each ALERT has the format
test-name_ALERT_alert-type_alert-level.
Click on the hyperlinks for more details of the test.

Alert level C

```
PLAT241_ALERT_2_C High   'MainMol' Ueq as Compared to Neighbors of       O007 Check
PLAT242_ALERT_2_C Low    'MainMol' Ueq as Compared to Neighbors of       Sr01 Check
```

Alert level G

```
PLAT004_ALERT_5_G Polymeric Structure Found with Maximum Dimension          1 Info
PLAT007_ALERT_5_G Number of Unrefined Donor-H Atoms .............           2 Report
              H00A  H00B
PLAT199_ALERT_1_G Reported _cell_measurement_temperature ..... (K)        273 Check
PLAT200_ALERT_1_G Reported   _diffrn_ambient_temperature ..... (K)        273 Check
PLAT299_ALERT_4_G Atom Site Occupancy Constrained at ............         0.5 Check
              H00A    H00B    H00D    H00E    H00C    H00F    H00G    H00H
PLAT720_ALERT_4_G Number of Unusual/Non-Standard Labels .........          18 Note
              Sr01    S002    S003    O004    O005    O006    O007    O008
              H00A    H00B    C009    H00D    H00E    H00C    C00A    H00F
              H00G    H00H
PLAT764_ALERT_4_G Overcomplete CIF Bond List Detected (Rep/Expd) .       1.27 Ratio
PLAT774_ALERT_1_G Check   X-Y Bond in CIF: Sr01     --Sr01      ..       4.56 Ang.
PLAT774_ALERT_1_G Check   X-Y Bond in CIF: Sr01     --Sr01      ..       4.56 Ang.
PLAT779_ALERT_4_G Suspect or Irrelevant (Bond) Angle(s) in CIF ...      40.77 Deg.
              O004 -S002 -SR01    4_565   1_555   3_666 ......     #   57 Check
PLAT779_ALERT_4_G Suspect or Irrelevant (Bond) Angle(s) in CIF ...      40.77 Deg.
              O004 -S002 -SR01    1_555   1_555   3_656 ......     #   59 Check
PLAT789_ALERT_4_G Atoms with Negative _atom_site_disorder_group  #          6 Check
PLAT822_ALERT_4_G CIF-embedded .res Contains Negative PART Numbers          2 Check
PLAT883_ALERT_1_G No Info/Value for _atom_sites_solution_primary .     Please Do !
```

0 **ALERT level A** = Most likely a serious problem - resolve or explain
0 **ALERT level B** = A potentially serious problem, consider carefully
2 **ALERT level C** = Check. Ensure it is not caused by an omission or oversight
14 **ALERT level G** = General information/check it is not something unexpected

5 ALERT type 1 CIF construction/syntax error, inconsistent or missing data
2 ALERT type 2 Indicator that the structure model may be wrong or deficient
0 ALERT type 3 Indicator that the structure quality may be low
7 ALERT type 4 Improvement, methodology, query or suggestion
2 ALERT type 5 Informative message, check

It is advisable to attempt to resolve as many as possible of the alerts in all categories. Often the minor alerts point to easily fixed oversights, errors and omissions in your CIF or refinement strategy, so attention to these fine details can be worthwhile. In order to resolve some of the more serious problems it may be necessary to carry out additional measurements or structure refinements. However, the purpose of your study may justify the reported deviations and the more serious of these should normally be commented upon in the discussion or experimental section of a paper or in the "special_details" fields of the CIF. checkCIF was carefully designed to identify outliers and unusual parameters, but every test has its limitations and alerts that are not important in a particular case may appear. Conversely, the absence of alerts does not guarantee there are no aspects of the results needing attention. It is up to the individual to critically assess their own results and, if necessary, seek expert advice.

Publication of your CIF in IUCr journals

A basic structural check has been run on your CIF. These basic checks will be run on all CIFs submitted for publication in IUCr journals (*Acta Crystallographica*, *Journal of Applied Crystallography*, *Journal of Synchrotron Radiation*); however, if you intend to submit to *Acta Crystallographica Section C* or *E* or *IUCrData*, you should make sure that full publication checks are run on the final version of your CIF prior to submission.

Publication of your CIF in other journals

Please refer to the *Notes for Authors* of the relevant journal for any special instructions relating to CIF submission.

PLATON version of 15/07/2024; check.def file version of 15/07/2024

Datablock 6 - ellipsoid plot

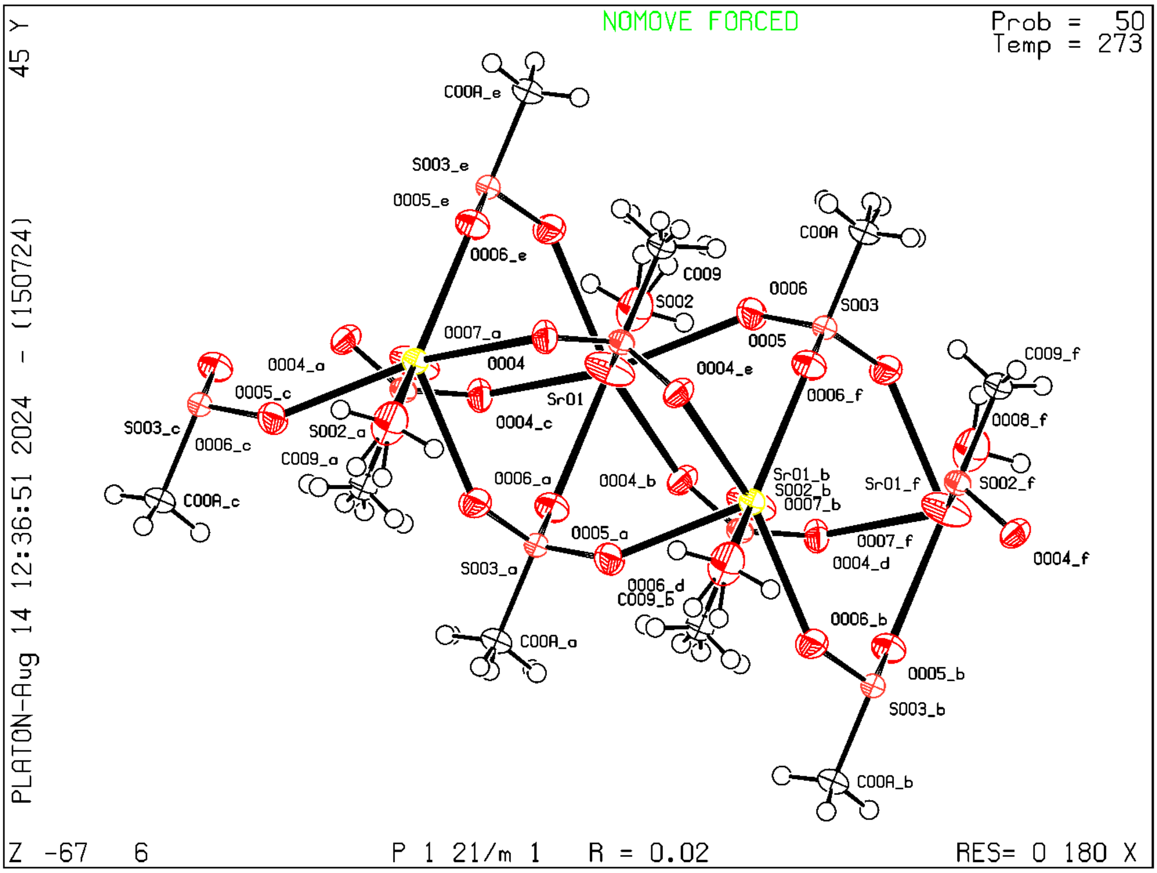

Supplement: Supplementary file 1 — oc4c01401_si_001.zip [file oc4c01401_si_001.zip › SrS2O7C2H8_checkcif.pdf]
