# Supplementary material for: The New Paradigm of Ligand Substitution-Driven Enhancement of Anisotropy from SO4 Units in Short-Wavelength Region
Source: ACS Cent Sci. 2024 Nov 27;10(12):2312–20. doi: 10.1021/acscentsci.4c01401 (PMC11673188; doi:10.1021/acscentsci.4c01401)
Supplement: Supplementary file 2 — oc4c01401_si_002.pdf [file oc4c01401_si_002.pdf]

The New Paradigm of Ligand Substitution - Driven Enhancement of Anisotropy from SO_4 Units in Short-Wavelength Region

Chenhui Hu,[a,b,#] Huimin Li,[a,#] Guangsheng Xu,[a,b] Zhihua Yang,[a,b] Jian Han[a,b,*] and Shilie Pan,[a,b,*]

[a] Research Center for Crystal Materials; State Key Laboratory of Functional Materials and Devices for Special Environmental Conditions; Xinjiang Key Laboratory of Functional Crystal Materials; Xinjiang Technical Institute of Physics and Chemistry, Chinese Academy of Sciences, 40-1 South Beijing Road, Urumqi 830011, China.

[b] Center of Materials Science and Optoelectronics Engineering, University of Chinese Academy of Sciences, Beijing 100049, China.

* Corresponding authors: hanjian@ms.xjb.ac.cn and slpan@ms.xjb.ac.cn.

[#] These authors contributed equally to this work.

EXPERIMENTAL METHODOLOGY

Experimental Methods

The crystal structures of most compounds were obtained by using acids and carbonates of the corresponding metals. The stoichiometric of MCO_3(M is the metal cation) or NH_3OHCl, and HSO_3CH_3/$HSO_3C_2H_5$/$HSO_3C_2H_4OH$ were dissolved and reacted in the deionized water at room temperature. The choice of carbonate allows us to visualize the reaction process through the bubbles produced. Then heating at about 50 °C and stirring well, so that they react well to obtain large-sized crystals, and grinding to obtain a polycrystalline powder of the target compounds. To obtain millimeter-sized crystals, we tried to make the cooling process slow at the rate of 1°C/day. In addition to this, vibrations to the surroundings are avoided as much as possible. Finally, the crystals needed can be obtained for the band gap in the transmission spectrum testing.

Synthesis.

The mild hydrothermal method with stoichiometric ratios was employed to get the crystals of $LiSO_3C_2H_4OH$, $LiSO_3C_2H_5\cdot H_2O$, $NaSO_3C_2H_4OH$, $KSO_3C_2H_4OH$, $CsH(SO_3CH_3)_2$, $(CN_4H_7)SO_3NH_2$, $Mg(SO_3CH_3)_2\cdot 2H_2O$, $Mn(SO_3CH_3)_2\cdot 2H_2O$, $NH_4SO_3CH_3$, $Sr(SO_3CH_3)_2\cdot H_2O$, $Zn(SO_3CH_3)_2\cdot 4H_2O$, $Zn(SO_3C_2H_4OH)_2\cdot 2H_2O$, $(NH_3OH)SO_3CH_3$, $Zn(SO_3C_2H_5)_2\cdot 6H_2O$, and $(CN_4H_7)S_2O_3$. The crystal structures of $Mg(SO_3CH_3)_2\cdot 2H_2O$, $Mn(SO_3CH_3)_2\cdot 2H_2O$, $NH_4SO_3CH_3$, $Sr(SO_3CH_3)_2\cdot H_2O$, $Zn(SO_3CH_3)_2\cdot 4H_2O$, and $Zn(SO_3C_2H_5)_2\cdot 6H_2O$ have been reported and our test results match them.[1-6]

Characterization.

The crystallographic data were collected on a Bruker D8 Venture X-ray single crystal diffractometer equipped with a PHOTON III C28 detector and integrated with the SAINT program[7] using monochromatic Mo Kα radiation or Cu Kα radiation at room temperature. Using the SHELXTL[8] crystallographic software package with the direct method completed the calculation, and PLATON[9] was used to confirm the final structure, and no other symmetries were found. All atoms were refined anisotropically,

and their positions were refined with full-matrix least-squares techniques. **Table S1** shows the information about the crystal structure refinement and data. **Table S2** shows the atomic coordinates and the equivalent isotropic displacement parameters, and **Tables S3 and S4** show the bond length and bond angles.

A Bruker D2 PHASER X-ray diffractometer was employed to characterize the powder XRD patterns with monochromatized Cu/Mo Kα radiation in the angular range of 2θ=5 (10) -70 ° for $LiSO_3C_2H_5 \cdot H_2O$, $NaSO_3C_2H_4OH$, $KSO_3C_2H_4OH$, $Mg(SO_3CH_3)_2 \cdot 2H_2O$, $NH_4SO_3CH_3$, $Sr(SO_3CH_3)_2 \cdot H_2O$, $Zn(SO_3CH_3)_2 \cdot 4H_2O$, $Zn(SO_3C_2H_4OH)_2 \cdot 2H_2O$, and $Zn(SO_3C_2H_5)_2 \cdot 6H_2O$, which was collected with the scan step width at 0.02 °.

The IR absorption spectra of the four compounds were recorded using a Shimadzu Affinity-1 Fourier transform IR spectrometer in the wavenumber range of 400-4000 cm^{-1}. The 5 mg of samples were mixed thoroughly with 500 mg of dried KBr and pressed into discs in a heat lamp. UV-vis-NIR diffuse-reflectance spectroscopy data were recorded using a Shimadzu 3700DUV spectrophotometer in the wavelength range from 180 to 2600 nm at room temperature.

The data of thermal behavior were collected by NETZSCH STA 449C simultaneous analyzer instrument under flowing nitrogen gas, and the sample and Al_2O_3 reference were put in closed platinum crucibles with a temperature from 40 to 600 ℃ at a heating rate of 10 ℃/ min.

Computational descriptions.

The first principles calculations for $LiSO_3C_2H_4OH$, $LiSO_3C_2H_5 \cdot H_2O$, $NaSO_3C_2H_4OH$, $KSO_3C_2H_4OH$, $CsH(SO_3CH_3)_2$, $(CN_4H_7)SO_3NH_2$, $Mg(SO_3CH_3)_2 \cdot 2H_2O$, $Mn(SO_3CH_3)_2 \cdot 2H_2O$, $NH_4SO_3CH_3$, $Sr(SO_3CH_3)_2 \cdot H_2O$, $Zn(SO_3CH_3)_2 \cdot 4H_2O$, $Zn(SO_3C_2H_4OH)_2 \cdot 2H_2O$, $(NH_3OH)SO_3CH_3$, $Zn(SO_3C_2H_5)_2 \cdot 6H_2O$, and $(CN_4H_7)S_2O_3$ were investigated using the plane wave density functional theory (DFT) package CASTEP.[10, 11] The exchange-correlation functional was treated by the generalized gradient approximation (GGA) in the formulation of Perdew-Burke-Emzerhoff (PBE) functional, and core-valence interactions were described by norm-conserving pseudopotentials (NCP).[12, 13] Because GGA usually

underestimates the bandgap owing to the discontinuity of exchange-correlation energy, the Heyd-Scuseria-Ernzerhof (HSE06) hybrid functional[14] was chosen to provide more accurate band gap values. The kinetic energy cutoffs, SCF tolerance, and the numerical integration of the Brillouin zone are shown in **Table S5**. Therefore, the bandgap difference between the GGA and HSE06 methods was used as the operation of a scissor to calculate optical properties. The default values of the CASTEP package were used on the aspect of the other calculation parameters and convergence criteria.

The orbitals and polarizability anisotropy of [$SO_2CH_3NH_2$], [SO_4F], [SO_3NH_2], [SO_4NH_3], [$SOCl(CH_3)$], [$SO(NH)(CH_3)_2$], [$SO_2(NH_2)(CH_3)_2$], [S_2O_3], [SO_3CH_3], [$SO_3C_2H_4OH$], and [$SO_3C_2H_5$] groups at the molecular level were calculated using DFT method implemented in the Gaussian09 package.[15] B3LYP (Becke, three-parameter, Lee-Yang-Parr) exchange-correlation functional[16] with the Lee–Yang–Parr correlation functional at the 6-31G basis set in Gaussian was employed.[17]

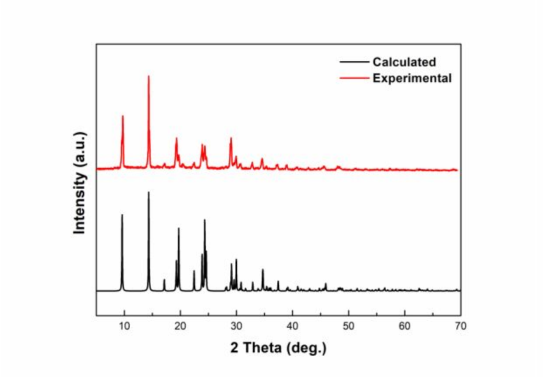
Calculated
Experimental
Intensity (a.u.)
10 20 30 40 50 60 70
2 Theta (deg.)

(a)

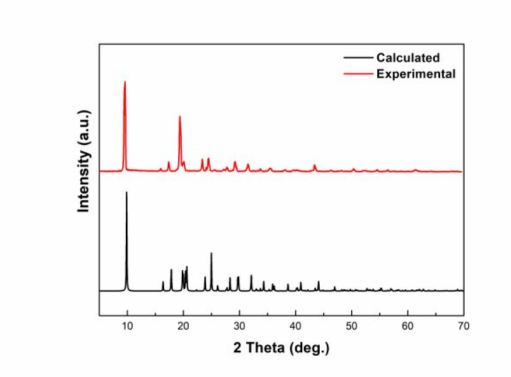
Calculated
Experimental
Intensity (a.u.)
10 20 30 40 50 60 70
2 Theta (deg.)

(b)

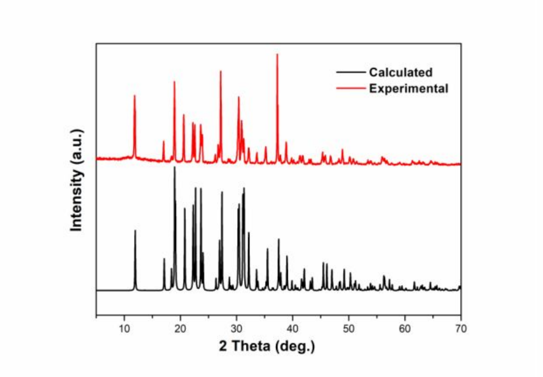
Calculated
Experimental
Intensity (a.u.)
10 20 30 40 50 60 70
2 Theta (deg.)

(c)

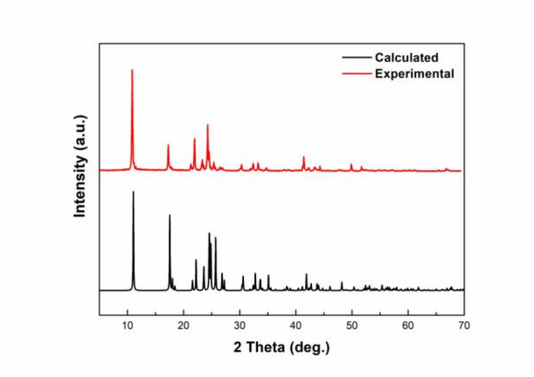
Calculated
Experimental
Intensity (a.u.)
10 20 30 40 50 60 70
2 Theta (deg.)

(d)

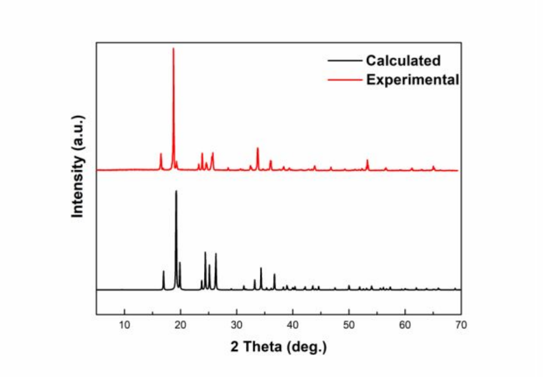
Calculated
Experimental
Intensity (a.u.)
10 20 30 40 50 60 70
2 Theta (deg.)

(e)

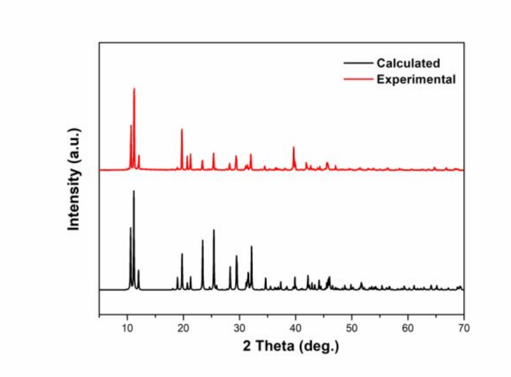
Calculated
Experimental
Intensity (a.u.)
10 20 30 40 50 60 70
2 Theta (deg.)

(f)

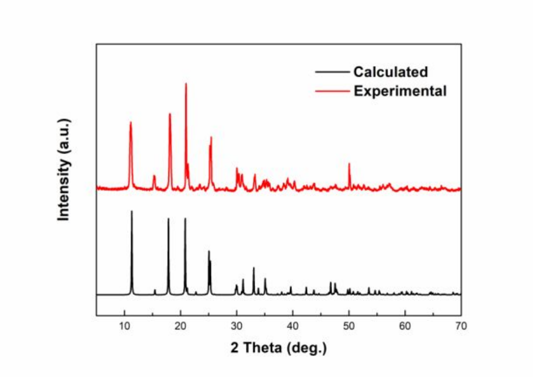

(g)

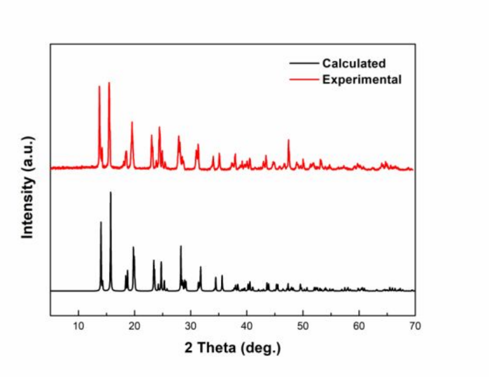

(h)

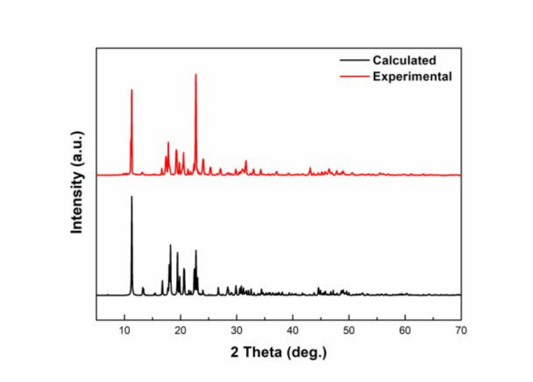

(i)

Fig. **S1** Experimental and calculated XRD patterns of (a) $LiSO_3C_2H_5{\cdot}H_2O$, (b) $NaSO_3C_2H_4OH$, (c) $KSO_3C_2H_4OH$, (d) $Mg(SO_3CH_3)_2{\cdot}2H_2O$, (e) $NH_4SO_3CH_3$, (f) $Sr(SO_3CH_3)_2{\cdot}H_2O$, (g) $Zn(SO_3CH_3)_2{\cdot}4H_2O$, (h) $Zn(SO_3C_2H_4OH)_2{\cdot}2H_2O$, and (i) $Zn(SO_3C_2H_5)_2{\cdot}6H_2O$.

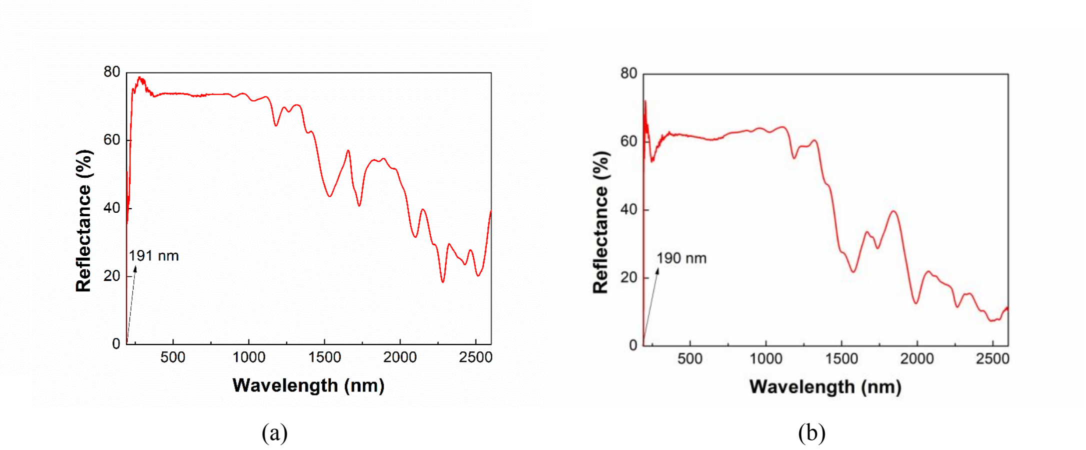

Fig. **S2** UV-vis-NIR diffuse reflectance spectra of (a) $KSO_3C_2H_4OH$, and (b) $Zn(SO_3C_2H_4OH)_2{\cdot}2H_2O$.

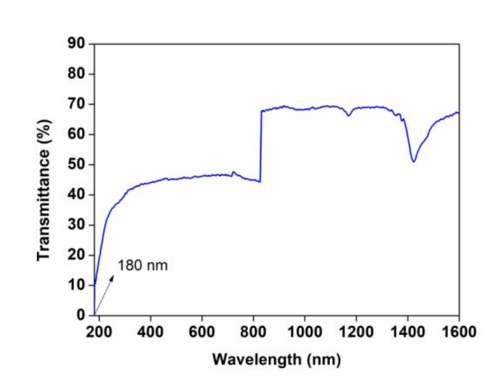

(a)

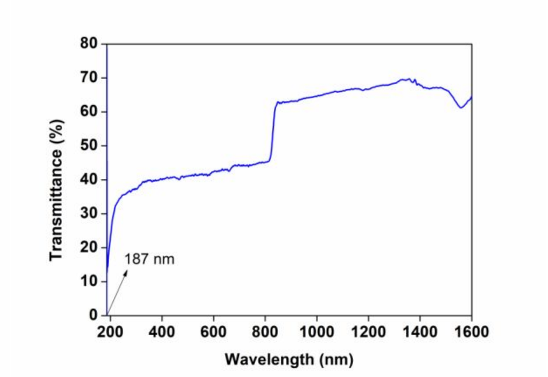

(b)

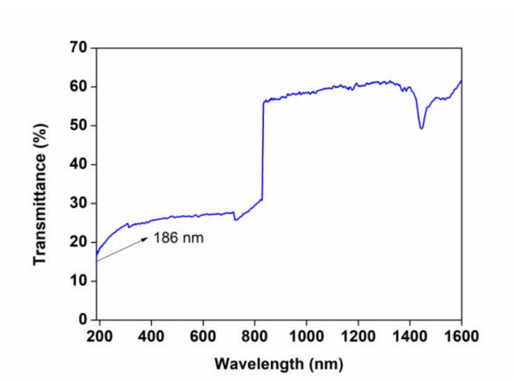

(c)

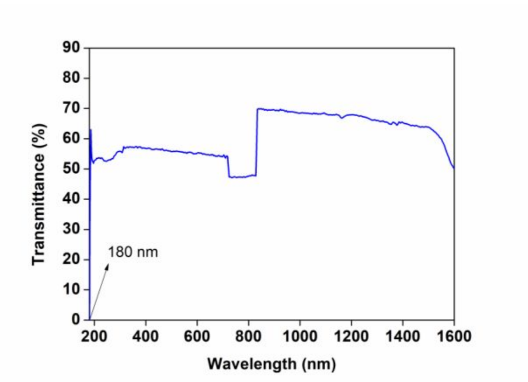

(d)

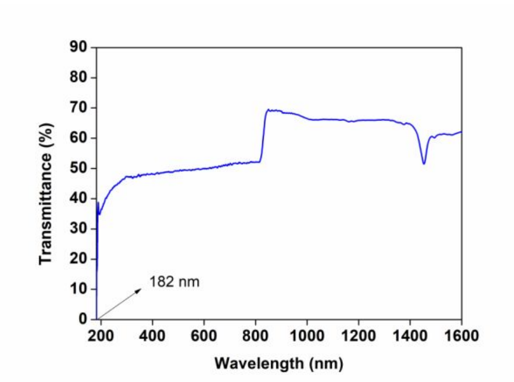

(e)

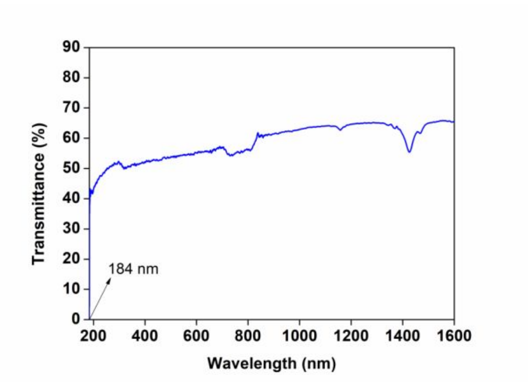

(f)

Fig. **S3** Transmittance spectra of (a) $LiSO_3C_2H_5{\cdot}H_2O$, (b) $NaSO_3C_2H_4OH$, (c) $Mg(SO_3CH_3)_2{\cdot}2H_2O$, (d) $NH_4SO_3CH_3$, (e) $Zn(SO_3CH_3)_2{\cdot}4H_2O$, and (f) $Sr(SO_3CH_3)_2{\cdot}H_2O$.

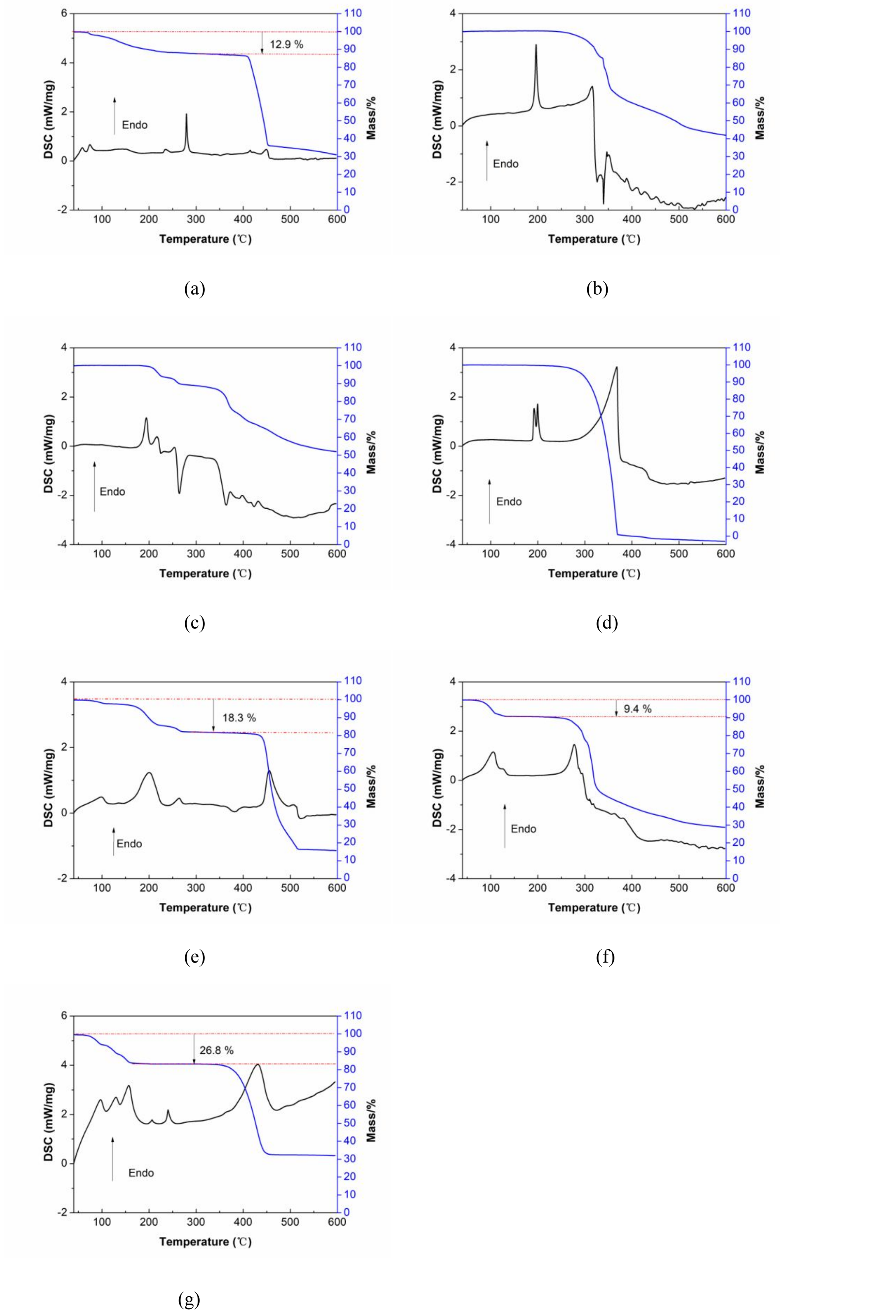

Fig. **S4** TG and DSC curves for (a) $LiSO_3C_2H_5{\cdot}H_2O$, (b) $NaSO_3C_2H_4OH$, (c) $KSO_3C_2H_4OH$, (d) $NH_4SO_3CH_3$, (e) $Mg(SO_3CH_3)_2{\cdot}2H_2O$, (f) $Zn(SO_3C_2H_4OH)_2{\cdot}2H_2O$, and (g) $Zn(SO_3C_2H_5)_2{\cdot}6H_2O$.

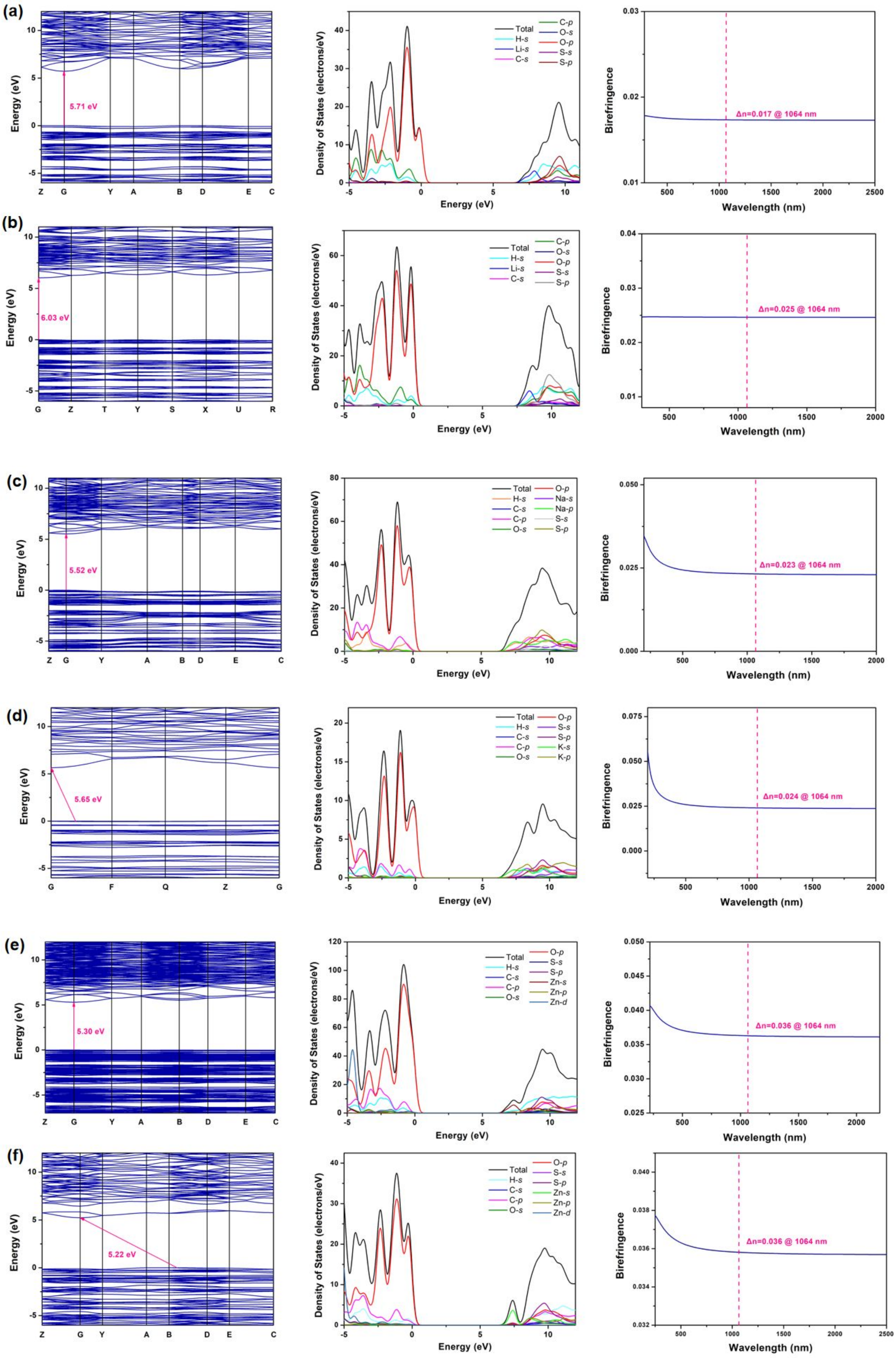
(a)
(b)
(c)
(d)
(e)
(f)
Energy (eV)
Density of States (electrons/eV)
Birefringence
Wavelength (nm)
5.71 eV
6.03 eV
5.52 eV
5.65 eV
5.30 eV
5.22 eV
Δn=0.017 @ 1064 nm
Δn=0.025 @ 1064 nm
Δn=0.023 @ 1064 nm
Δn=0.024 @ 1064 nm
Δn=0.036 @ 1064 nm
Δn=0.036 @ 1064 nm
Total
H-s
Li-s
C-s
C-p
O-s
O-p
S-s
S-p
Na-s
Na-p
K-s
K-p
Zn-s
Zn-p
Zn-d

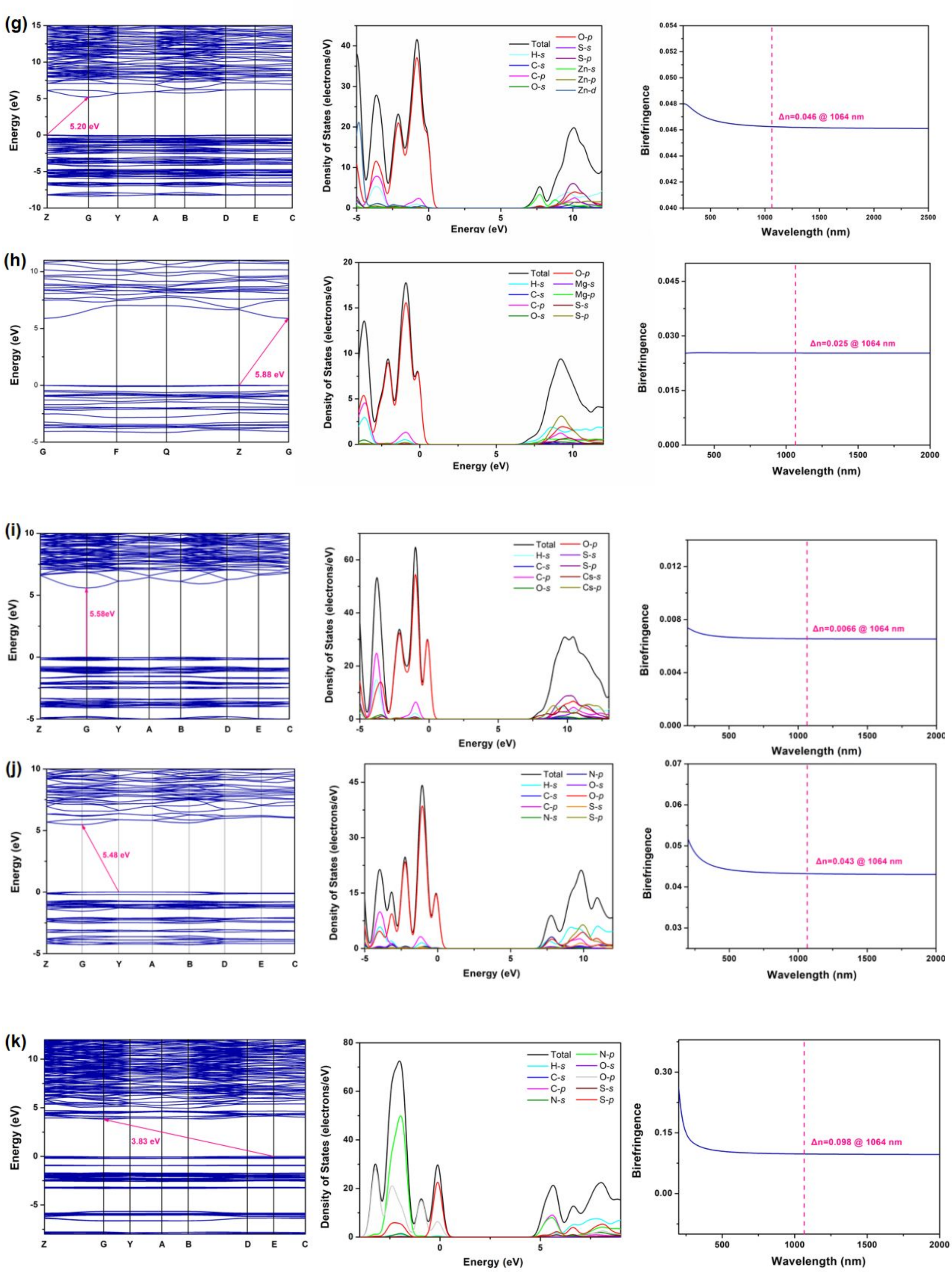

Fig. **S5** Electronic structures, partial density of states, and calculated birefringence of (a) $LiSO_3C_2H_5·H_2O$, (b) $LiSO_3CH_2CH_2OH$, (c) $NaSO_3CH_2CH_2OH$, (d) $KSO_3CH_2CH_2OH$, (e) $Zn(SO_3C_2H_5)_2·6H_2O$, (f) $Zn(SO_3CH_2CH_2OH)_2·2H_2O$, (g) $Zn(SO_3CH_3)_2·4H_2O$, (h) $Mg(SO_3CH_3)_2·2H_2O$, (i) $CsHSO_3CH_3$, (j) $(NH_3OH)SO_3CH_3$, and (k) $(CN_4H_7)_2S_2O_3$.

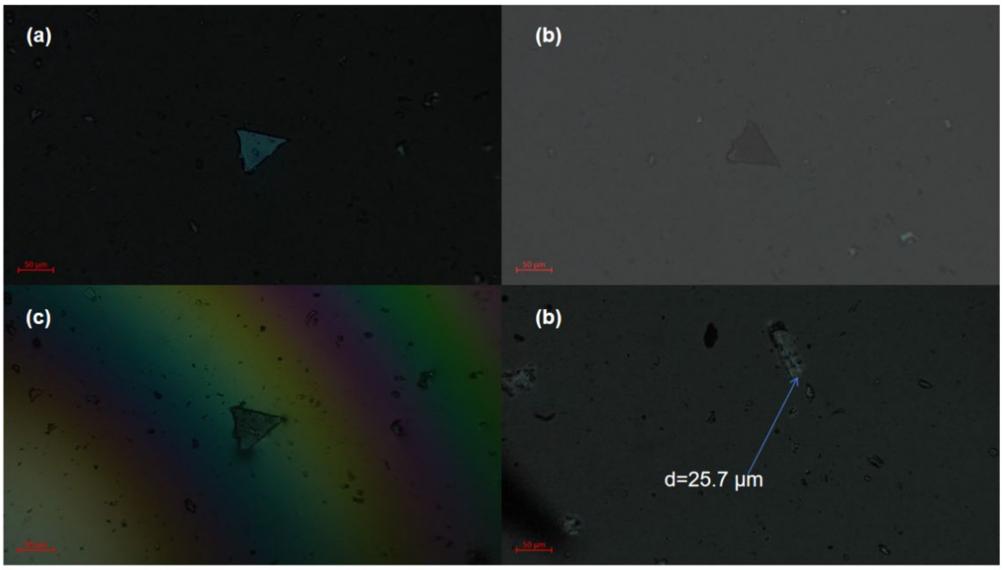

Fig. **S6** The assessment of the difference in refractive index of $NH_4SO_3CH_3$ by polarizing microscopy. (a) Single crystals under the polarizing microscope. (b) The extinction state of crystals. (c) The positive rotation of compensatory. (d) The thickness of the crystal.

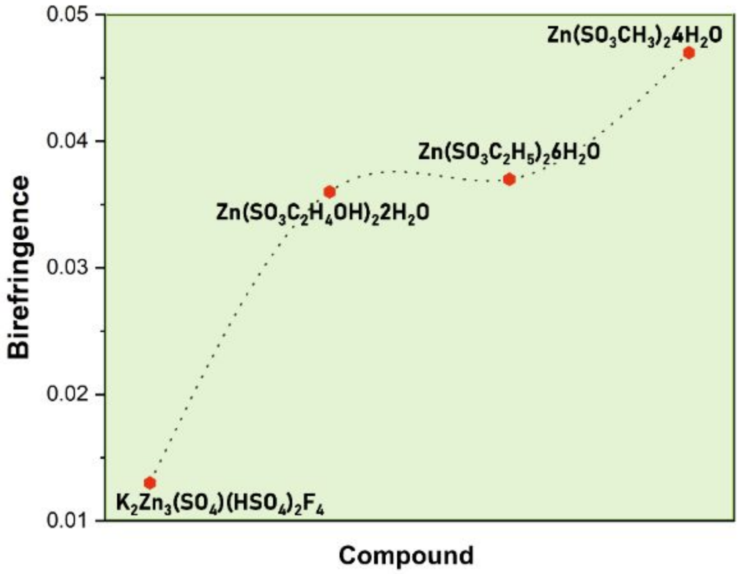

Fig. **S7** The sulfates containing zinc get the gains in birefringence by the design and modification of the [SO_4] groups.

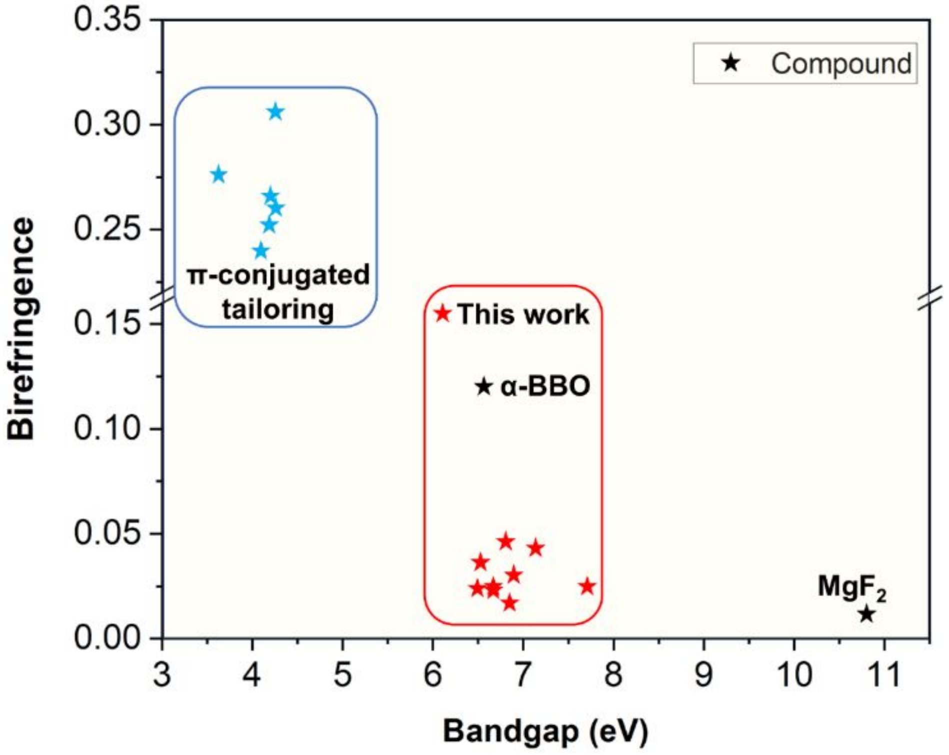

Fig. **S8** The comparison of the present work with sulfate crystals through π-conjugated tailoring and commercial birefringent crystals, where black stars represent commercialized crystals, red stars represent this work, and blue stars represent crystals through π-conjugated tailoring.

Table S1. Crystal data and structure refinements for $LiSO_3C_2H_4OH$, $LiSO_3C_2H_5 \cdot H_2O$, $NaSO_3C_2H_4OH$, $KSO_3C_2H_4OH$, $CsH(SO_3CH_3)_2$, $(CN_4H_7)SO_3NH_2$, $(CN_4H_7)S_2O_3$, $Mg(SO_3CH_3)_2 \cdot 2H_2O$, $Mn(SO_3CH_3)_2 \cdot 2H_2O$, $NH_4SO_3CH_3$, $Sr(SO_3CH_3)_2 \cdot H_2O$, $Zn(SO_3CH_3)_2 \cdot 4H_2O$, $Zn(SO_3C_2H_4OH)_2 \cdot 2H_2O$, $(NH_3OH)SO_3CH_3$, and $Zn(SO_3C_2H_5)_2 \cdot 6H_2O$.

**Empirical formula**	**$LiSO_3C_2H_4OH$**	**$LiSO_3C_2H_5 \cdot H_2O$**	**$NaSO_3C_2H_4OH$**
**Temperature** (K)		298(2)	
**Crystal system**	orthorhombic	monoclinic	monoclinic
**Space group**	*Pbcn*	$P2_1/n$	*C2/c*
**Formula weight**	380.23	134.08	148.11
a **(Å)**	10.0567(2)	10.9561(8)	6.9410(2)
b **(Å)**	9.7653(2)	5.1614(4)	8.8712(3)
c **(Å)**	10.3804(3)	11.1821(8)	18.1958(6)
**α (°)**	90	90	90
**β (°)**	90	112.325(3)	100.648(2)
**γ (°)**	90	90	90
**Z, Volume (Å^3)**	8, 1019.42(4)	4, 584.94(8)	8, 1101.11(6)
**ρ_{Calcd} (mg/m^3)**	1.721	1.522	1.787
**μ(/mm)**	5.003	4.360	5.456
**F(000)**	544.0	280.0	608.0
**R(int)**	0.0559	0.0831	0.0571
**Goodness-of-fit on F^2**	1.091	1.150	1.112
**Final R indices**	$R_1 = 0.0229$,	$R_1 = 0.0442$,	$R_1 = 0.0404$,
**[F_o 2>2σ(F_o^2)]^a**	$wR_2 = 0.0672$	$wR_2 = 0.1097$	$wR_2 = 0.1038$
**R indices (all data)^a**	$R_1 = 0.0256$,	$R_1 = 0.0456$,	$R_1 = 0.0482$,
	$wR_2 = 0.0683$	$wR_2 = 0.1110$	$wR_2 = 0.1093$
**Largest diff. peak and hole (e·Å^{-3})**	0.263 / -0.336	0.314 / -0.683	0.546 / -0.565

[a] $R_1 = \sum||F_o| - |F_c||/\sum|F_o|$ and $wR_2 = [\sum w(F_o^2 - F_c^2)^2/\sum wF_o^4]^{1/2}$ for $F_o^2 > 2\sigma(F_o^2)$.

Empirical formula	$KSO_3C_2H_4OH$	$CsH(SO_3CH_3)_2$	$(CN_4H_7)SO_3NH_2$
**Temperature**		298(2) K	
**Crystal system**	triclinic	monoclinic	Orthorhombic
**Space group**	$P\bar{1}$	$C2/c$	$Pca2_1$
**Formula weight**	164.22	324.11	171.19
a **(Å)**	5.8146(5)	13.4638(5)	13.4926(12)
b **(Å)**	6.6190(5)	9.9042(3)	7.5386(8)
c **(Å)**	7.4934(6)	8.8318(6)	6.8623(8)
α **(º)**	95.298(4)	90	90
β **(º)**	96.167(4)	129.9320(10)	90
γ **(º)**	98.507(4)	90	90
Z**, Volume (Å^3)**	2, 281.85(4)	4, 903.07(8)	4, 698.00(13)
ρ_{Calcd} **(mg/m^3)**	1.935	2.384	1.629
μ**(/mm)**	11.190	4.547	0.427
F**(000)**	168.0	616.0	360.0
R**(int)**	0.0653	0.0771	0.0914
**Goodness-of-fit on** F^2	1.103	1.025	1.105
**Flack parameter**	/	/	0.06(9)
**Final *R* indices**	R_1 = 0.0425,	R_1 = 0.0192,	R_1 = 0.0387,
**[F_o 2>2σ(F_o^2)]^a**	wR_2 = 0.1315	wR_2 = 0.0433	wR_2 = 0.0764
R **indices (all data)^a**	R_1 = 0.0488,	R_1 = 0.0218,	R_1 = 0.0529,
	wR_2 = 0.1361	wR_2= 0.0440	wR_2 = 0.0878
**Largest diff. peak and hole (e·Å^{-3})**	0.489 / -0.673	0.466 / -0.528	0.227 / -0.254

[a] $R_1 = \sum||F_o| - |F_c||/\sum|F_o|$ and $wR_2 = [\sum w(F_o^2 - F_c^2)^2/\sum wF_o^4]^{1/2}$ for $F_o^2>2\sigma(F_o^2)$.

Empirical formula	$(CN_4H_7)S_2O_3$	$Mg(SO_3CH_3)_2·2H_2O$	$Mn(SO_3CH_3)_2·2H_2O$
**Temperature**		298(2) K	
**Crystal system**	monoclinic	triclinic	triclinic
**Space group**	$C2/m$	$P\bar{1}$	$P\bar{1}$
**Formula weight**	262.33	250.53	281.16
a **(Å)**	12.8413(3)	5.10940(10)	5.157(3)
b **(Å)**	13.8848(4)	5.59400(10)	5.687(3)
c **(Å)**	6.8825(2)	8.4891(2)	8.545(5)
α **(º)**	90	77.6720(10)	77.91(2)
β **(º)**	115.408(2)	74.5920(10)	74.92(2)
γ **(º)**	90	89.1500(10)	89.03(2)
Z**, Volume ($Å^3$)**	4, 1108.45(5)	1, 228.295(8)	1, 236.5(2)
**ρ_{Calcd} (mg/m^3)**	1.572	1.822	1.975
**μ(/mm)**	4.484	6.206	1.850
F**(000)**	552.0	130.0	143.0
R**(int)**	0.0943	0.0496	0.0778
**Goodness-of-fit on F^2**	1.049	1.136	1.104
**Final *R* indices**	R_1 = 0.0375,	R_1 = 0.0283,	R_1 = 0.0398,
**[F_o 2>2σ(F_o^2)][a]**	wR_2 = 0.0969	wR_2 = 0.0865	wR_2 = 0.0998
R **indices (all data)[a]**	R_1 = 0.0489,	R_1 = 0.0298,	R_1 = 0.0456,
	wR_2 = 0.1010	wR_2 = 0.0877	wR_2 = 0.1070
**Largest diff. peak and hole ($e·Å^{-3}$)**	0.356 / -0.403	0.405 / -0.384	0.925 / -0.654

[a] $R_1 = \sum||F_o| - |F_c||/\sum|F_o|$ and $wR_2 = [\sum w(F_o^2 - F_c^2)^2/\sum wF_o^4]^{1/2}$ for $F_o^2>2\sigma(F_o^2)$.

**Empirical formula**	**$NH_4SO_3CH_3$**	**$Sr(SO_3CH_3)_2·H_2O$**	**$Zn(SO_3CH_3)_2·4H_2O$**
**Temperature**		298(2) K	
**Crystal system**	monoclinic	monoclinic	monoclinic
**Space group**	*C*2/*m*	*P*2$_1$/*m*	*P*2$_1$/c
**Formula weight**	113.14	295.82	327.62
a **(Å)**	7.4927(7)	8.6144(9)	8.0215(14)
b **(Å)**	7.2826(7)	6.0625(6)	9.9274(18)
c **(Å)**	9.2278(9)	9.0776(11)	7.2147(13)
α **(º)**	90	90	90
β **(º)**	93.372(5)	113.126(4)	103.154(9)
γ **(º)**	90	90	90
***Z*, Volume (Å^3)**	4, 502.66(8)	2, 435.98(8)	2, 559.45(17)
**ρ_{Calcd} (mg/m^3)**	1.495	2.253	1.945
**μ(/mm)**	0.529	6.661	6.956
***F*(000)**	240.0	292.0	336.0
***R*(int)**	0.0551	0.0560	0.0695
**Goodness-of-fit on F^2**	1.164	1.171	1.144
**Final *R* indices**	R_1 = 0.0438,	R_1 = 0.0203,	R_1 = 0.0347,
**[F_o 2>2σ(F_o^2)]^a**	wR_2 = 0.1300	wR_2 = 0.0480	wR_2 = 0.1029
***R* indices (all data)^a**	R_1 = 0.0452,	R_1 = 0.0225,	R_1 = 0.0388,
	wR_2 = 0.1314	wR_2 = 0.0486	wR_2 = 0.1060
**Largest diff. peak and hole (e·Å^{-3})**	0.533 and -0.641	0.450 / -0.399	0.722 / -0.711

[a] $R_1 = \sum||F_o| - |F_c||/\sum|F_o|$ and $wR_2 = [\sum w(F_o^2 - F_c^2)^2/\sum wF_o^4]^{1/2}$ for $F_o^2 > 2\sigma(F_o^2)$.

Empirical formula	$Zn(SO_3C_2H_4OH)_2{\cdot}2H_2O$	$(NH_3OH)SO_3CH_3$	$Zn(SO_3C_2H_5)_2{\cdot}6H_2O$
**Temperature**		298(2) K	
**Crystal system**	monoclinic	monoclinic	monoclinic
**Space group**	$P2_1$/n	$P2_1/c$	$P2_1/c$
**Formula weight**	351.64	129.14	391.74
a **(Å)**	6.313(2)	8.6687(7)	12.561(5)
b **(Å)**	12.366(4)	7.6256(5)	9.777(4)
c **(Å)**	7.336(3)	8.1545(7)	12.989(6)
α **(º)**	90	90	90
β **(º)**	90.508(10)	112.220(4)	91.10(2)
γ **(º)**	90	90	90
Z**, Volume (Å^3)**	2, 572.7(4)	4, 499.02(7)	4, 1595.0(12)
**ρ_{Calcd} (mg/m^3)**	2.039	1.719	1.631
**μ(/mm)**	2.551	0.559	1.849
F**(000)**	360.0	272.0	816.0
R**(int)**	0.0839	0.0628	0.1323
**Goodness-of-fit on F^2**	1.035	1.040	1.095
**Final *R* indices [F_o 2>2σ(F_o^2)]^a**	R_1 = 0.0563, wR_2 = 0.1154	R_1 = 0.0255, wR_2 = 0.0694	R_1 = 0.0549, wR_2 = 0.1274
***R* indices (all data)^a**	R_1 = 0.0928, wR_2 = 0.1465	R_1 = 0.0306, wR_2 = 0.0730	R_1 = 0.0803, wR_2 = 0.1589
**Largest diff. peak and hole (e·Å^{-3})**	0.789 / -0.735	0.249 / -0.348	0.987 / -0.548

[a] $R_1 = \sum||F_o| - |F_c||/\sum|F_o|$ and $wR_2 = [\sum w(F_o^2 - F_c^2)^2/\sum wF_o^4]^{1/2}$ for $F_o^2>2\sigma(F_o^2)$.

Table S2a. Fractional atomic coordinates (×10^4) and equivalent isotropic displacement parameters (Å^2×10^3) for $LiSO_3C_2H_4OH$. U_{eq} is defined as 1/3 of the trace of the orthogonalised U_{ij} tensor.

**Atom**	***x***	***y***	***z***	***U*(eq)**
S(1)	3643(1)	4279(1)	6692(1)	18(1)
O(1)	2945(1)	4630(1)	7870(1)	34(1)
O(2)	2813(1)	4446(1)	5541(1)	29(1)
O(3)	4883(1)	5026(1)	6569(1)	26(1)
O(4)	6088(1)	2504(1)	5597(1)	39(1)
C(1)	4041(2)	2514(2)	6807(1)	28(1)
C(2)	4785(2)	1948(2)	5668(2)	32(1)
Li(1)	1560(3)	4413(3)	9092(3)	25(1)

Table S2b. Fractional atomic coordinates (×10[4]) and equivalent isotropic displacement parameters (Å[2]×10[3]) for $LiSO_3C_2H_5 \cdot H_2O$. U_{eq} is defined as 1/3 of the trace of the orthogonalised U_{ij} tensor.

**Atom**	***x***	***y***	***z***	***U*(eq)**
S(1)	3915(1)	2097(1)	5896(1)	28(1)
O(1)	4059(2)	-591(3)	6308(2)	44(1)
O(3)	5080(2)	3606(3)	6589(2)	45(1)
O(2)	3497(2)	2318(3)	4496(2)	45(1)
O(4)	6949(2)	7639(4)	8390(2)	51(1)
C(2)	2615(3)	3406(5)	6272(2)	43(1)
C(1)	1288(3)	2169(6)	5519(3)	59(1)
Li(1)	5591(4)	7188(7)	6705(3)	34(1)

Table S2c. Fractional atomic coordinates (×10^4) and equivalent isotropic displacement parameters (Å^2×10^3) for $NaSO_3C_2H_4OH$. U_{eq} is defined as 1/3 of the trace of the orthogonalised U_{ij} tensor.

**Atom**	***x***	***y***	***z***	***U*(eq)**
Na(1)	5000	1138(2)	7500	31(1)
Na(2)	5000	7272(2)	7500	28(1)
O(1)	6722(4)	5529(3)	6924(2)	50(1)
O(2)	4044(3)	4309(4)	6122(2)	54(1)
O(3)	6622(4)	2856(3)	6860(2)	54(1)
O(4)	10524(3)	4174(3)	6590(1)	31(1)
C(1)	7448(6)	4297(7)	5731(2)	65(2)
C(2)	9424(6)	3935(9)	5888(2)	86(2)
S(1)	6090(1)	4238(1)	6465(1)	24(1)

Table S2d. Fractional atomic coordinates (×10^4) and equivalent isotropic displacement parameters (Å^2×10^3) for $KSO_3C_2H_4OH$. U_{eq} is defined as 1/3 of the trace of the orthogonalised U_{ij} tensor.

**Atom**	***x***	***y***	***z***	***U*(eq)**
K(1)	2000(1)	2996(1)	9961(1)	42(1)
S(1)	2616(1)	7908(1)	7193(1)	25(1)
O(1)	2085(6)	5777(4)	7525(4)	47(1)
O(2)	1135(5)	9198(4)	8036(4)	45(1)
O(3)	5093(4)	8725(4)	7660(3)	42(1)
O(4)	3026(5)	7050(4)	1856(3)	42(1)
C(1)	1962(6)	7974(5)	4827(4)	30(1)
C(2)	3455(7)	6781(5)	3724(4)	34(1)

Table S2e. Fractional atomic coordinates (×10^4) and equivalent isotropic displacement parameters (Å^2×10^3) for $CsH(SO_3CH_3)_2$. U_{eq} is defined as 1/3 of the trace of the orthogonalised U_{ij} tensor.

**Atom**	***x***	***y***	***z***	***U*(eq)**
Cs(1)	5000	5255(1)	7500	37(1)
S(1)	2760(1)	2195(1)	2829(1)	31(1)
O(1)	3115(2)	3534(2)	3607(3)	55(1)
O(2)	1430(2)	1859(2)	1771(3)	48(1)
O(3)	3131(2)	1929(2)	1575(3)	65(1)
C(1)	3729(3)	1058(3)	4763(4)	44(1)

Table S2f. Fractional atomic coordinates (×10^4) and equivalent isotropic displacement parameters (Å^2×10^3) for $(CN_4H_7)SO_3NH_2$. U_{eq} is defined as 1/3 of the trace of the orthogonalised U_{ij} tensor.

**Atom**	***x***	***y***	***z***	***U*(eq)**
S(1)	8671(1)	837(1)	4852(2)	35(1)
O(1)	9031(2)	2566(4)	5430(6)	54(1)
O(2)	7615(2)	859(4)	4465(5)	50(1)
O(3)	9243(3)	77(5)	3312(7)	59(1)
C(1)	6892(3)	5616(5)	5593(7)	40(1)
N(1)	7851(3)	5852(5)	5707(6)	50(1)
N(2)	6266(3)	6953(5)	5784(7)	50(1)
N(3)	6546(3)	4012(4)	5276(7)	50(1)
N(4)	5521(3)	3725(6)	5189(11)	68(2)
N(5)	8767(3)	-542(5)	6670(7)	43(1)

Table S2g. Fractional atomic coordinates ($\times 10^4$) and equivalent isotropic displacement parameters ($Å^2 \times 10^3$) for $Zn(SO_3C_2H_4OH)_2 \cdot 2H_2O$. U_{eq} is defined as 1/3 of the trace of the orthogonalised U_{ij} tensor.

**Atom**	***x***	***y***	***z***	***U*(eq)**
Zn(1)	5000	5000	5000	31(1)
S(1)	4751(2)	3106(1)	2040(2)	31(1)
O(3)	5681(7)	3527(4)	3719(6)	38(1)
O(5)	5046(7)	1938(4)	1934(7)	47(1)
O(1)	2019(7)	5025(4)	3732(6)	38(1)
O(2)	6267(8)	5839(4)	2869(7)	47(1)
O(4)	5504(7)	3671(4)	468(6)	46(1)
C(1)	1996(10)	3308(5)	2183(9)	37(2)
C(2)	1315(11)	4483(5)	2117(9)	38(2)

Table S2h. Fractional atomic coordinates ($\times 10^4$) and equivalent isotropic displacement parameters ($Å^2 \times 10^3$) for $(NH_3OH)SO_3CH_3$. U_{eq} is defined as 1/3 of the trace of the orthogonalised U_{ij} tensor.

**Atom**	***x***	***y***	***z***	***U*(eq)**
S(1)	2424(1)	1486(1)	5270(1)	22(1)
O(1)	1234(1)	2938(2)	4860(2)	31(1)
O(2)	2409(2)	441(2)	6743(2)	43(1)
O(3)	2175(2)	439(1)	3693(2)	33(1)
C(1)	4409(2)	2429(2)	5920(3)	37(1)
O(4)	2268(2)	6318(2)	5287(2)	36(1)
N(1)	1250(2)	6929(2)	3599(2)	27(1)

Table S2i. Fractional atomic coordinates (×10^4) and equivalent isotropic displacement parameters (Å^2×10^3) for $(CN_4H_7)S_2O_3$. U_{eq} is defined as 1/3 of the trace of the orthogonalised U_{ij} tensor.

Atom	*x*	*y*	*z*	*U*(eq)
S(1)	2663(1)	5000	6166(1)	29(1)
S(2)	1480(1)	5000	3113(2)	48(1)
O(1)	2547(2)	5873(1)	7254(3)	44(1)
O(2)	3799(2)	5000	6090(5)	40(1)
N(1)	3836(2)	7565(2)	7135(4)	44(1)
N(2)	5563(2)	8233(2)	7629(4)	46(1)
N(3)	5362(2)	6589(2)	7672(4)	39(1)
N(4)	6516(2)	6473(2)	7992(4)	47(1)
C(1)	4927(2)	7464(2)	7480(4)	33(1)

Table S3a. Bond lengths for $LiSO_3C_2H_4OH$.

Atom	Length/Å	Atom	Length/Å
S(1)-O(1)	1.4507(11)	O(4)-H(4)	0.82
S(1)-O(2)	1.4662(10)	C(1)-H(1A)	0.97
S(1)-O(3)	1.4504(11)	C(1)-H(1B)	0.97
S(1)-C(1)	1.7735(16)	C(1)-C(2)	1.505(2)
Li(1)-O(1)	1.895(3)	C(2)-H(2A)	0.97
Li(1)#1-O(2)	1.976(3)	C(2)-H(2B)	0.97
Li(1)#2-O(3)	1.917(3)	C(2)-O(4)	1.4203(19)
Li(1)#2-O(4)	1.951(3)		

Symmetry transformations used to generate equivalent atoms:

#1 -x+1/2,-y+1,z-1/2 #2 x+1/2,y,-z+3/2 #3 -x+1/2,-y+1,z+1/2

#4 x-1/2,y,-z+3/2

Table S3b. Bond lengths for $LiSO_3C_2H_5 \cdot H_2O$.

Atom	Length/Å	Atom	Length/Å
S(1)-O(1)	1.4514(15)	C(1)-H(2)	0.96
S(1)-O(2)	1.4586(17)	C(1)-H(3)	0.96
S(1)-O(3)	1.4444(16)	C(2)-C(1)	1.516(4)
S(1)-C(2)	1.765(2)	C(2)-H(4)	0.97
Li(1)#1-O(1)	1.939(4)	C(2)-H(7)	0.97
Li(1)#2-O(2)	1.972(4)	O(4)-H(5)	0.8538
Li(1)-O(3)	1.921(4)	O(4)-H(6)	0.8537
Li(1)-O(4)	1.920(4)		
C(1)-H(1)	0.96		

Symmetry transformations used to generate equivalent atoms:

#1 x,y-1,z #2 -x+1,-y+1,-z+1 #3 x,y+1,z

Table S3c. Bond lengths for $NaSO_3C_2H_4OH$.

Atom	Length/Å	Atom	Length/Å
S(1)-C(1)	1.773(4)	Na(2)-O(3)#4	2.472(3)
S(1)-O(1)	1.437(3)	Na(2)-O(3)#2	2.472(3)
S(1)-O(2)	1.444(2)	Na(2)-O(4)#2	2.438(3)
S(1)-O(3)	1.434(3)	Na(2)-O(4)#4	2.438(3)
Na(1)-O(1)#6	2.385(3)	C(1)-H(1)	0.97
Na(1)-O(1)#7	2.385(3)	C(1)-H(2)	0.97
Na(1)-O(3)	2.330(3)	C(1)-C(2)	1.386(6)
Na(1)-O(3)#5	2.330(3)	C(2)-H(3)	0.97
Na(1)-O(4)#6	2.477(3)	C(2)-H(4)	0.97
Na(1)-O(4)#7	2.477(3)	O(4)-H(5)	0.98
Na(2)-O(1)#5	2.319(3)	O(4)-C(2)	1.378(5)
Na(2)-O(1)	2.319(3)		

Symmetry transformations used to generate equivalent atoms:

#1 x+1/2,y+1/2,z   #2 x-1/2,y+1/2,z   #3 x,y+1,z

#4 -x+3/2,y+1/2,-z+3/2   #5 -x+1,y,-z+3/2   #6 x-1/2,y-1/2,z

#7 -x+3/2,y-1/2,-z+3/2   #8 x+1/2,y-1/2,z   #9 x,y-1,z

Table S3d. Bond lengths for $KSO_3C_2H_4OH$.

Atom	Length/Å	Atom	Length/Å
K(1)-O(1)	2.708(2)	S(1)-O(2)	1.451(3)
K(1)-O(1)#1	3.332(3)	S(1)-O(3)	1.454(3)
K(1)-O(2)#3	2.730(3)	S(1)-C(1)	1.779(3)
K(1)-O(2)#1	2.809(3)	O(4)-H(5)	0.946(10)
K(1)-O(3)#2	2.762(3)	O(4)-C(2)	1.427(4)
K(1)-O(4)#4	3.329(3)	C(1)-H(1)	0.97
K(1)-O(4)#5	2.866(3)	C(1)-H(2)	0.97
K(1)-O(4)#6	3.083(3)	C(1)-C(2)	1.516(5)
K(1)-C(2)#5	3.535(4)	C(2)-H(3)	1.00(5)
S(1)-O(1)	1.449(3)	C(2)-H(4)	0.98

Symmetry transformations used to generate equivalent atoms:

#1 -x,-y+1,-z+2 #2 -x+1,-y+1,-z+2 #3 x,y-1,z

#4 -x+1,-y+1,-z+1 #5 x,y,z+1 #6 -x,-y+1,-z+1

#7 x,y+1,z #8 x,y,z-1

Table S3e. Bond lengths for $CsH(SO_3CH_3)_2$.

**Atom**	**Length/Å**	**Atom**	**Length/Å**
Cs(1)-O(1)#1	3.1568(19)	Cs(1)-O(3)#4	3.741(3)
Cs(1)-O(1)#2	3.467(2)	Cs(1)-O(3)#3	3.484(2)
Cs(1)-O(1)	3.1568(19)	S(1)-O(1)	1.4268(19)
Cs(1)-O(1)#3	3.467(2)	S(1)-O(2)	1.4290(18)
Cs(1)-O(2)#4	3.337(2)	S(1)-O(3)	1.5025(19)
Cs(1)-O(2)#5	3.1775(19)	S(1)-C(1)	1.740(2)
Cs(1)-O(2)#6	3.1775(19)	O(3)-H(4)	1.208(2)
Cs(1)-O(2)#7	3.337(2)	C(1)-H(1)	0.96
Cs(1)-O(3)#2	3.484(3)	C(1)-H(2)	0.96
Cs(1)-O(3)#7	3.741(2)	C(1)-H(3)	0.96

Symmetry transformations used to generate equivalent atoms:

#1 -x+1,y,-z+3/2 #2 -x+1,-y+1,-z+1 #3 x,-y+1,z+1/2

#4 x+1/2,y+1/2,z+1 #5 -x+1/2,-y+1/2,-z+1 #6 x+1/2,-y+1/2,z+1/2

#7 -x+1/2,y+1/2,-z+1/2 #8 x-1/2,y-1/2,z-1

Table S3f. Bond lengths for $(CN_4H_7)SO_3NH_2$.

Atom	Length/Å	Atom	Length/Å
S(1)-O(1)	1.446(3)	C(1)-N(3)	1.315(5)
S(1)-O(2)	1.450(3)	N(3)-H(5)	0.86
S(1)-O(3)	1.428(4)	N(3)-N(4)	1.400(6)
S(1)-N(5)	1.629(4)	N(4)-H(4)	0.8616
N(1)-H(7)	0.86	N(4)-H(3)	0.89(11)
N(1)-H(6)	0.86	N(5)-H(9)	0.80(5)
N(2)-H(1)	0.86	N(5)-H(8)	0.98(6)
N(2)-H(2)	0.86		
C(1)-N(1)	1.309(5)		
C(1)-N(2)	1.322(5)		

Table S3g. Bond lengths for $Zn(SO_3C_2H_4OH)_2{\cdot}2H_2O$.

Atom	Length/Å	Atom	Length/Å
Zn(1)-O(1)#1	2.092(4)	O(1)-H(2)	0.82
Zn(1)-O(1)	2.092(4)	O(1)-C(2)	1.429(7)
Zn(1)-O(2)#1	2.045(5)	O(2)-H(1)	0.8501
Zn(1)-O(2)	2.045(5)	O(2)-H(2)	0.8501
Zn(1)-O(3)#1	2.096(4)	C(1)-H(5)	0.97
Zn(1)-O(3)	2.096(4)	C(1)-H(6)	0.97
S(1)-O(3)	1.456(4)	C(1)-C(2)	1.516(9)
S(1)-O(5)	1.458(5)	C(2)-H(4)	0.97
S(1)-O(4)	1.433(5)	C(2)-H(3)	0.97
S(1)-C(1)	1.761(7)		

Symmetry transformations used to generate equivalent atoms:

#1 -x+1,-y+1,-z+1

Table S3h. Bond lengths for $(NH_3OH)SO_3CH_3$.

Atom	Length/Å	Atom	Length/Å
S(1)-O(1)	1.4635(11)	C(1)-H(3)	0.9800
S(1)-O(2)	1.4453(12)	O(4)-H(7)	0.8400
S(1)-O(3)	1.4582(11)	O(4)-N(1)	1.4038(17)
S(1)-C(1)	1.7521(17)	N(1)-H(5)	0.8846
C(1)-H(1)	0.9800	N(1)-H(6)	0.8847
C(1)-H(2)	0.9800	N(1)-H(4)	0.91(2)

Table S3i. Bond lengths for $(CN_4H_7)S_2O_3$.

Atom	Length/Å	Atom	Length/Å
S(1)-S(2)	1.9965(14)	N(2)-H(4)	0.86
S(1)-O(1)#1	1.4657(17)	N(2)-C(1)	1.321(3)
S(1)-O(1)	1.4657(17)	N(3)-H(5)	0.86
S(1)-O(2)	1.481(2)	N(3)-N(4)	1.411(3)
N(1)-H(1)	0.86	N(3)-C(1)	1.320(3)
N(1)-H(2)	0.86	N(4)-H(6)	0.8716
N(1)-C(1)	1.324(3)		
N(2)-H(3)	0.86		

Symmetry transformations used to generate equivalent atoms:

#1 x,-y+1,z

Table S4a. Bond angles for $LiSO_3C_2H_4OH$.

Atom	Angle/°	Atom	Angle/°
O(1)-S(1)-O(2)	112.63(7)	O(1)-Li(1)-O(2)#1	102.28(13)
O(1)-S(1)-C(1)	106.39(7)	O(1)-Li(1)-O(3)#2	111.83(14)
O(2)-S(1)-C(1)	106.92(7)	O(1)-Li(1)-O(4)#2	113.35(14)
O(3)-S(1)-O(1)	111.83(7)	O(3)#2-Li(1)-O(2)#1	112.16(13)
O(3)-S(1)-O(2)	111.21(6)	O(3)#2-Li(1)-O(4)#2	98.23(12)
O(3)-S(1)-C(1)	107.49(7)	O(4)#2-Li(1)-O(2)#1	119.39(14)

Symmetry transformations used to generate equivalent atoms:

#1 -x+1/2,-y+1,z+1/2 #2 x-1/2,y,-z+3/2

Table S4b. Bond angles for $LiSO_3C_2H_5 \cdot H_2O$.

Atom	Angle/°	Atom	Angle/°
O(1)-S(1)-O(2)	111.54(10)	H(2)-C(1)-H(1)	109.5
O(1)-S(1)-C(2)	106.92(10)	O(1)#2-Li(1)-O(2)#1	112.68(18)
O(3)-S(1)-O(1)	112.18(10)	O(3)-Li(1)-O(1)#2	110.42(19)
O(3)-S(1)-O(2)	112.74(10)	O(3)-Li(1)-O(2)#1	106.74(19)
O(3)-S(1)-C(2)	107.13(11)	O(4)-Li(1)-O(1)#2	115.7(2)
O(2)-S(1)-C(2)	105.85(11)	O(4)-Li(1)-O(3)	106.28(18)
H(6)-O(4)-H(5)	104.3	O(4)-Li(1)-O(2)#1	104.36(19)
H(4)-C(2)-H(7)	107.7		
H(3)-C(1)-H(2)	109.5		
H(3)-C(1)-H(1)	109.5		

Symmetry transformations used to generate equivalent atoms:

#1 -x+1,-y+1,-z+1 #2 x,y+1,z

Table S4c. Bond angles for $NaSO_3C_2H_4OH$.

Atom	Angle/°	Atom	Angle/°
O(1)-S(1)-O(2)	112.76(17)	O(1)#3-Na(2)-O(4)#2	90.04(9)
O(1)-S(1)-C(1)	105.5(2)	O(1)#3-Na(2)-O(4)#1	157.23(9)
O(3)-S(1)-O(1)	111.53(19)	O(1)-Na(2)-O(4)#1	90.04(9)
O(3)-S(1)-O(2)	113.31(18)	O(1)-Na(2)-O(4)#2	157.23(9)
O(3)-S(1)-C(1)	106.2(2)	O(1)#3-Na(2)-O(1)	96.36(16)
O(2)-S(1)-C(1)	106.83(17)	O(1)#3-Na(2)-O(3)#1	80.18(9)
O(3)#3-Na(1)-O(4)#4	159.78(8)	O(1)-Na(2)-O(3)#1	116.76(11)
O(3)-Na(1)-O(4)#4	88.96(9)	O(1)-Na(2)-O(3)#2	80.18(9)
O(3)-Na(1)-O(4)#5	159.78(8)	O(1)#3-Na(2)-O(3)#2	116.76(11)
O(3)#3-Na(1)-O(4)#5	88.96(9)	O(3)#2-Na(2)-O(3)#1	155.79(15)
O(3)#3-Na(1)-O(1)#4	81.83(9)	O(4)#2-Na(2)-O(4)#1	92.43(12)
O(3)-Na(1)-O(1)#4	116.04(11)	O(4)#2-Na(2)-O(3)#2	77.44(8)
O(3)-Na(1)-O(1)#5	81.82(9)	O(4)#1-Na(2)-O(3)#2	85.83(10)
O(3)#3-Na(1)-O(1)#5	116.04(11)	O(4)#2-Na(2)-O(3)#1	85.83(10)
O(3)-Na(1)-O(3)#3	98.31(16)	O(4)#1-Na(2)-O(3)#1	77.44(8)
O(4)#4-Na(1)-O(4)#5	90.57(12)	H(1)-C(1)-H(2)	107.1
O(1)#4-Na(1)-O(4)#4	78.04(8)	H(3)-C(2)-H(4)	106.8
O(1)#4-Na(1)-O(4)#5	83.57(10)		
O(1)#5-Na(1)-O(4)#5	78.04(8)		
O(1)#5-Na(1)-O(4)#4	83.57(10)		
O(1)#4-Na(1)-O(1)#5	153.77(15)		

Symmetry transformations used to generate equivalent atoms:

#1 x-1/2,y+1/2,z #2 -x+3/2,y+1/2,-z+3/2

#3 -x+1,y,-z+3/2 #4 x-1/2,y-1/2,z

#5 -x+3/2,y-1/2,-z+3/2

Table S4d. Bond angles for $KSO_3C_2H_4OH$.

Atom	Angle/°	Atom	Angle/°
O(1)-K(1)-O(1)#1	103.31(7)	O(3)#2-K(1)-O(1)#1	102.61(8)
O(1)-K(1)-O(2)#3	106.77(8)	O(3)#2-K(1)-O(2)#1	76.76(8)
O(1)-K(1)-O(2)#1	140.38(9)	O(3)#2-K(1)-O(4)#5	95.45(8)
O(1)-K(1)-O(3)#2	141.96(9)	O(3)#2-K(1)-O(4)#4	73.92(7)
O(1)-K(1)-O(4)#5	71.09(8)	O(3)#2-K(1)-O(4)#6	147.08(8)
O(1)-K(1)-O(4)#4	73.34(8)	O(4)#5-K(1)-O(1)#1	65.38(7)
O(1)-K(1)-O(4)#6	70.84(8)	O(4)#4-K(1)-O(1)#1	163.64(7)
O(2)#3-K(1)-O(1)#1	116.84(8)	O(4)#6-K(1)-O(1)#1	60.59(7)
O(2)#1-K(1)-O(1)#1	45.34(7)	O(4)#5-K(1)-O(4)#6	101.22(7)
O(2)#3-K(1)-O(2)#1	78.19(9)	O(4)#5-K(1)-O(4)#4	98.73(7)
O(2)#3-K(1)-O(3)#2	85.40(8)	O(4)#6-K(1)-O(4)#4	130.09(8)
O(2)#3-K(1)-O(4)#5	177.42(8)	O(1)-S(1)-O(2)	112.82(18)
O(2)#1-K(1)-O(4)#5	104.37(8)	O(1)-S(1)-O(3)	111.97(17)
O(2)#3-K(1)-O(4)#4	79.14(8)	O(2)-S(1)-O(3)	112.16(17)
O(2)#1-K(1)-O(4)#4	144.04(7)		
O(2)#3-K(1)-O(4)#6	79.21(8)		
O(2)#1-K(1)-O(4)#6	71.71(7)		

Symmetry transformations used to generate equivalent atoms:

#1 -x,-y+1,-z+2 #2 -x+1,-y+1,-z+2 #3 x,y-1,z

#4 -x+1,-y+1,-z+1 #5 x,y,z+1 #6 -x,-y+1,-z+1

Table S4e. Bond angles for $CsH(SO_3CH_3)_2$.

Atom	Angle/°	Atom	Angle/°
O(1)#1-Cs(1)-O(1)	114.63(7)	O(2)#5-Cs(1)-O(1)#3	61.47(5)
O(1)#2-Cs(1)-O(1)#3	139.51(6)	O(2)#5-Cs(1)-O(2)#6	97.57(7)
O(1)-Cs(1)-O(1)#3	105.77(6)	O(2)#4-Cs(1)-O(2)#7	123.14(7)
O(1)-Cs(1)-O(1)#2	95.85(5)	O(2)#6-Cs(1)-O(2)#7	93.99(5)
O(1)#1-Cs(1)-O(1)#3	95.85(5)	O(2)#5-Cs(1)-O(2)#4	93.99(5)
O(1)#1-Cs(1)-O(1)#2	105.77(6)	O(2)#6-Cs(1)-O(2)#4	123.90(6)
O(1)-Cs(1)-O(2)#4	164.88(5)	O(2)#5-Cs(1)-O(2)#7	123.90(6)
O(1)-Cs(1)-O(2)#5	72.35(5)	O(2)#4-Cs(1)-O(3)#2	63.12(4)
O(1)-Cs(1)-O(2)#6	65.89(5)	O(2)#7-Cs(1)-O(3)#3	63.12(4)
O(1)#1-Cs(1)-O(2)#7	164.88(5)	O(2)#7-Cs(1)-O(3)#7	39.23(4)
O(1)#1-Cs(1)-O(2)#4	63.18(5)	O(2)#7-Cs(1)-O(3)#2	71.93(5)
O(1)-Cs(1)-O(2)#7	63.18(5)	O(2)#6-Cs(1)-O(3)#7	123.53(5)
O(1)#1-Cs(1)-O(2)#5	65.89(5)	O(2)#5-Cs(1)-O(3)#4	123.53(5)
O(1)#1-Cs(1)-O(2)#6	72.35(5)	O(2)#6-Cs(1)-O(3)#2	97.74(5)
O(1)#2-Cs(1)-O(3)#7	98.75(5)	O(2)#6-Cs(1)-O(3)#4	91.81(5)
O(1)#2-Cs(1)-O(3)#4	62.68(4)	O(2)#5-Cs(1)-O(3)#7	91.81(5)
O(1)#3-Cs(1)-O(3)#2	101.88(5)	O(2)#7-Cs(1)-O(3)#4	110.65(5)
O(1)#2-Cs(1)-O(3)#3	101.88(5)	O(2)#4-Cs(1)-O(3)#7	110.65(5)
O(1)#1-Cs(1)-O(3)#7	155.32(5)	O(2)#5-Cs(1)-O(3)#3	97.74(5)
O(1)#1-Cs(1)-O(3)#4	64.53(5)	O(2)#5-Cs(1)-O(3)#2	156.96(5)
O(1)#3-Cs(1)-O(3)#3	40.54(4)	O(2)#6-Cs(1)-O(3)#3	156.96(5)
O(1)#3-Cs(1)-O(3)#7	62.68(4)	O(2)#4-Cs(1)-O(3)#3	71.93(5)
O(1)-Cs(1)-O(3)#4	155.32(5)	O(2)#4-Cs(1)-O(3)#4	39.23(4)
O(1)#2-Cs(1)-O(3)#2	40.54(4)	O(3)#2-Cs(1)-O(3)#3	73.62(7)
O(1)#3-Cs(1)-O(3)#4	98.75(5)	O(3)#3-Cs(1)-O(3)#7	38.87(7)
O(1)#1-Cs(1)-O(3)#3	129.97(5)	O(3)#2-Cs(1)-O(3)#7	93.96(3)
O(1)-Cs(1)-O(3)#7	64.53(5)	O(3)#2-Cs(1)-O(3)#4	38.87(7)
O(1)-Cs(1)-O(3)#3	102.84(5)	O(3)#3-Cs(1)-O(3)#4	93.96(3)
O(1)#1-Cs(1)-O(3)#2	102.84(5)	O(3)#4-Cs(1)-O(3)#7	127.39(7)
O(1)-Cs(1)-O(3)#2	129.97(5)	O(1)-S(1)-O(2)	115.07(13)
O(2)#5-Cs(1)-O(1)#2	159.00(5)	O(1)-S(1)-O(3)	110.55(14)
O(2)#6-Cs(1)-O(1)#3	159.00(5)	O(2)-S(1)-O(3)	110.32(13)
O(2)#7-Cs(1)-O(1)#3	99.11(5)	H(1)-C(1)-H(2)	109.5
O(2)#6-Cs(1)-O(1)#2	61.47(5)	H(1)-C(1)-H(3)	109.5
O(2)#4-Cs(1)-O(1)#3	60.80(5)	H(2)-C(1)-H(3)	109.5
O(2)#4-Cs(1)-O(1)#2	99.11(5)		
O(2)#7-Cs(1)-O(1)#2	60.80(5)		

Symmetry transformations used to generate equivalent atoms:

#1 -x+1,y,-z+3/2 #2 -x+1,-y+1,-z+1 #3 x,-y+1,z+1/2 #4 x+1/2,y+1/2,z+1 #5 -x+1/2,-y+1/2,-z+1 #6 x+1/2,-y+1/2,z+1/2 #7 -x+1/2,y+1/2,-z+1/2

Table S4f. Bond angles for $(CN_4H_7)SO_3NH_2$.

**Atom**	**Angle/°**	**Atom**	**Angle/°**
O(1)-S(1)-O(2)	111.70(17)	O(3)-S(1)-N(5)	105.5(2)
O(1)-S(1)-N(5)	109.8(2)	N(3)-C(1)-N(2)	119.4(4)
O(2)-S(1)-N(5)	103.0(2)	N(1)-C(1)-N(3)	119.1(4)
O(3)-S(1)-O(1)	112.5(2)	N(1)-C(1)-N(2)	121.5(4)
O(3)-S(1)-O(2)	113.6(2)		

Table S4g. Bond angles for $Zn(SO_3C_2H_4OH)_2{\cdot}2H_2O$.

Atom	Angle/°	Atom	Angle/°
O(1)#1-Zn(1)-O(3)	89.95(17)	O(2)-Zn(1)-O(1)#1	89.49(19)
O(1)#1-Zn(1)-O(3)#1	90.05(17)	O(2)-Zn(1)-O(2)#1	180
O(1)-Zn(1)-O(3)#1	89.95(17)	O(3)-Zn(1)-O(3)#1	180
O(1)-Zn(1)-O(3)	90.05(17)	O(3)-S(1)-O(5)	110.4(3)
O(1)#1-Zn(1)-O(1)	180.0(2)	O(3)-S(1)-C(1)	106.8(3)
O(2)#1-Zn(1)-O(3)#1	90.9(2)	O(5)-S(1)-C(1)	105.7(3)
O(2)-Zn(1)-O(3)	90.9(2)	O(4)-S(1)-O(3)	111.8(3)
O(2)-Zn(1)-O(3)#1	89.1(2)	O(4)-S(1)-O(5)	113.4(3)
O(2)#1-Zn(1)-O(3)	89.1(2)	O(4)-S(1)-C(1)	108.3(3)
O(2)#1-Zn(1)-O(1)	89.49(19)	H(1)-O(2)-H(2)	104.5
O(2)#1-Zn(1)-O(1)#1	90.51(19)	H(5)-C(1)-H(6)	107.6
O(2)-Zn(1)-O(1)	90.51(19)	H(4)-C(2)-H(3)	108.2

Symmetry transformations used to generate equivalent atoms:

#1 -x+1,-y+1,-z+1

Table S4h. Bond angles for $(NH_3OH)SO_3CH_3$.

Atom	Angle/°	Atom	Angle/°
O(1)-S(1)-C(1)	106.58(8)	H(1)-C(1)-H(2)	109.5
O(2)-S(1)-O(1)	112.30(8)	H(1)-C(1)-H(3)	109.5
O(2)-S(1)-O(3)	112.74(8)	H(2)-C(1)-H(3)	109.5
O(2)-S(1)-C(1)	106.95(9)	H(5)-N(1)-H(6)	108.9
O(3)-S(1)-O(1)	110.85(7)	H(5)-N(1)-H(4)	113.0
O(3)-S(1)-C(1)	107.00(8)	H(6)-N(1)-H(4)	109.5

Table S4i. Bond angles for $(CN_4H_7)S_2O_3$.

Atom	Angle/°	Atom	Angle/°
O(1)-S(1)-S(2)	109.83(9)	H(3)-N(2)-H(4)	120
O(1)#1-S(1)-S(2)	109.83(9)	H(6)-N(4)-H(7)	108.2
O(1)#1-S(1)-O(1)	111.62(16)	N(2)-C(1)-N(1)	120.0(2)
O(1)#1-S(1)-O(2)	109.59(9)	N(3)-C(1)-N(1)	119.0(2)
O(1)-S(1)-O(2)	109.59(9)	N(3)-C(1)-N(2)	121.0(2)
O(2)-S(1)-S(2)	106.23(12)		
H(1)-N(1)-H(2)	120		

Symmetry transformations used to generate equivalent atoms:

#1 x,-y+1,z

Table S5. The kinetic energy cutoffs, SCF tolerance, and the numerical integration of the Brillouin zone for compounds.

Compound	Energy cutoff (eV)	SCF tolerance (eV/atom)	K-points
$Zn(SO_3CH_3)_2 \cdot 4H_2O$	750	1.0×10^{-6}	3×2×5
$Zn(SO_3C_2H_4OH)_2 \cdot 2H_2O$	750	1.0×10^{-6}	3×2×5
$NaSO_3C_2H_4OH$	750	1.0×10^{-6}	3×2×5
$KSO_3C_2H_4OH$	830	5.0×10^{-7}	3×3×2
$Mg(SO_3CH_3)_2 \cdot 2H_2O$	990	5.0×10^{-7}	4×2×2
$Zn(SO_3C_2H_5)_2 \cdot 6H_2O$	830	5.0×10^{-7}	4×3×2
$LiSO_3C_2H_4OH$	830	5.0×10^{-7}	4×4×4
$LiSO_3C_2H_5 \cdot H_2O$	830	5.0×10^{-7}	4×4×2
$(NH_3OH)SO_3CH_3$	830	5.0×10^{-7}	4×3×2
$CsH(SO_3CH_3)_2$	830	5.0×10^{-7}	4×3×2
$(CN_4H_7)SO_3NH_2$	830	5.0×10^{-7}	3×4×4
$(CN_4H_7)S_2O_3$	830	5.0×10^{-7}	3×2×5

Table S6. The real-space atom-cutting analysis of the birefringence was used for $(CN_4H_7)SO_3NH_2$. The cutting radius of C, N, S, O, and H were set as 0.24, 1.10, 0.37, 1.11 and 0.29.

Unit	Birefringence contribution
SO_3NH_2	0.032
CN_4H_7	0.143

References

(1) Singh, R.; Kociok-Köhn, G.; Jassal, A. K.; Singh, L. Three-dimensional Hydrogen-bonded Magnesium (II) Supramolecular Motifs Based on in Situ Generated Alkanesulfonate (Me/Et/nPrSO_3^-) Ligands: A Combined Experimental and Computational Study. *Polyhedron* **2020**, 175, 114200.

(2) Frank, W.; Wallus, S. First Concluding Crystallographic Characterization of Zinc Alkanesulfonates. *Z. Anorg. Allg. Chem.* **2008**, 634, 2038.

(3) Đorđević, M.; Jeremić, D.; Kaluđerović, G. N.; Gómez-Ruiz, S.; Anđelković, B.; Radanović, D.; Brč eski, I. Synthesis and Spectroscopic Properties of Large Single-Crystals of Pb (II), Hg (II), and Sr (II) Methanesulfona to 1D Coordination Polymers. *Polyhedron* **2014**, 80, 282.

(4) Wei, C. Structure of Ammonium Methanesulfonate. *Acta Crystallogr. C* **1986**, 42, 1839.

(5) Belova, E. V.; Shakirova, J. D.; Lyssenko, K. A.; Mikheev, I. V.; Maliutin, A. S.; Kovalenko, N. A.; Uspenskaya, I. A. Phase Equilibria, Structural and Thermodynamic Properties of Phases in the Nickel (II) Methanesulfonate – Water, Cobalt (II) Methanesulfonate – Water and Manganese (II) Methanesulfonate – Water Systems. *J. Chem. Thermodynamics*, **2023**, 182, 107049.

(6) Russell, V. A.; Etter, M. C.; Ward, M. D. Layered Materials by Molecular Design: Structural Enforcement by Hydrogen Bonding in Guanidinium Alkane- and Arenesulfonates. *J. Am. Chem. Soc.* **1994**, 116, 1941.

(7) SAINT, V8.40B; Bruker Nano, Inc., **2019**.

(8) Sheldrick, G. M. SHELXTL; Bruker AXS Inc.: Madison, WI, **2008**.

(9) Spek, A. L. Single-crystal Structure Validation with the Program PLATON. *J. Appl. Crystallogr.* **2003**, 36, 7.

(10) Clark, S. J.; Segall, M. D.; Pickard, C. J.; Hasnip, P. J.; Probert, M. I.; Refson, K.; Payne, M. C. First Principles Methods Using CASTEP. *Z. Kristallogr. - Cryst. Mater.* **2005**, 220, 567.

(11) Kohn, W. Nobel Lecture: Electronic Structure of Matterwave Functions and Density Functionals. *Rev. Mod. Phys.* **1999**, 71, 1253.

(12) Perdew, J. P.; Burke, K.; Ernzerhof, M. Generalized Gradient Approximation Made Simple. *Phys. Rev. Lett.* **1996**, 77, 3865.

(13) Rappe, A. M.; Rabe, K. M.; Kaxiras, E.; Joannopoulos, J. D. Optimized Pseudopotentials. *Phys. Rev. B* **1990**, 41, 1227.

(14) Heyd, J.; Scuseria, G. E.; Ernzerhof, M. Hybrid Functionals Based on a Screened Coulomb Potential. *J. Chem. Phys.* **2003**, 118, 8207-8215.

(15) Frisch, M. J.; Trucks, G. W.; Schlegel, H. B.; Scuseria, G. E.; Robb, M. A.; Cheeseman, J. R.; Scalmani, G.; Barone, V.; Mennucci, B.; Petersson, G. A.; Nakatsuji, H.; Caricato, M.; Li, X.; Hratchian, H. P.; Izmaylov, A. F.; Bloino, J.; Zheng, G.; Sonnenberg, J. L.; Hada, M.; Ehara, M.; Toyota, K.; Fukuda, R.; Hasegawa, J.; Ishida, M.; Nakajima, T.; Honda, Y.; Kitao, O.; Nakai, H.; Vreven, T.; Montgomery, J. A.; Jr.; Peralta, J. E.; Ogliaro, F.; Bearpark, M.; Heyd, J. J.; Brothers, E.; Kudin, K. N.; Staroverov, V. N.; Kobayashi, R.; Normand, J.; Raghavachari, K.; Rendell, A.; Burant, J. C.; Iyengar, S. S.; Tomasi, J.; Cossi, M.; Rega, N.; Millam, J. M.; Klene, M.; Knox, J. E.; Cross, J. B.; Bakken, V.; Adamo, C.; Jaramillo, J.; Gomperts, R.; Stratmann, R. E.; Yazyev, O.; Austin, A. J.; Cammi, R.; Pomelli, C.; Ochterski, J. W.; Martin, R. L.; Morokuma, K.; Zakrzewski, V. G.; Voth, G. A.; Salvador, P.; Dannenberg, J. J.; Dapprich, S.; Daniels, A. D.; Farkas, O.; Foresman, J. B.; Ortiz, J. V.; Cioslowski, J.; Fox, D. J.; Gaussian 09, Revision A.02, Gaussian, Inc., Wallingford CT **2009**.

(16) Stephens, P. J.; Devlin, F. J.; Chabalowski, C. F.; Frisch, M. J. Ab Initio Calculation of Vibrational Absorption and Circular Dichroism Spectra Using Density Functional Force Fields. *J. Phys. Chem.* **1994**, 98, 11623−11627.

(17) Hehre, W. J.; Ditchfield, R.; Pople, J. A. Self-Consistent Molecular-Orbital Methods. IX. An Extended Gaussian-Type Basis for Molecular-Orbital Studies of Organic Molecules. *J. Chem. Phys.* **1972**, 56, 2257.
